# Targeting PARP-1 and DNA Damage Response Defects in Colorectal Cancer Chemotherapy with Established and Novel PARP Inhibitors

**DOI:** 10.3390/cancers16203441

**Published:** 2024-10-10

**Authors:** Philipp Demuth, Lea Thibol, Anna Lemsch, Felix Potlitz, Lukas Schulig, Christoph Grathwol, Georg Manolikakes, Dennis Schade, Vassilis Roukos, Andreas Link, Jörg Fahrer

**Affiliations:** 1 Department of Chemistry, Division of Food Chemistry and Toxicology, RPTU Kaiserslautern-Landau, 67663 Kaiserslautern, Germany; pdemuth@rptu.de (P.D.); thibol@rptu.de (L.T.); anna.lemsch@gmx.de (A.L.); 2 Department of Pharmaceutical and Medicinal Chemistry, Institute of Pharmacy, University of Greifswald, 17489 Greifswald, Germany; felix.potlitz@gmail.com (F.P.); lukas.schulig@uni-greifswald.de (L.S.); link@uni-greifswald.de (A.L.); 3 Institute of Biological and Chemical Systems—Functional Molecular Systems (IBCS-FMS), Karlsruhe Institute of Technology (KIT), 76344 Eggenstein-Leopoldshafen, Germany; christoph.grathwol@kit.edu; 4 Department of Chemistry, RPTU Kaiserslautern-Landau, 67663 Kaiserslautern, Germany; manolikakes@chemie.uni-kl.de; 5 Department of Pharmaceutical and Medicinal Chemistry, Institute of Pharmacy, Christian-Albrechts-University of Kiel, 24118 Kiel, Germany; schade@pharmazie.uni-kiel.de; 6 Institute of Molecular Biology, 55128 Mainz, Germany; v.roukos@imb-mainz.de

**Keywords:** PARP-1, colorectal cancer, synthetic lethality, DNA damage response, chemotherapy

## Abstract

**Simple Summary:**

Inhibition of the DNA repair protein PARP-1 is a promising concept in cancer therapy. More recently, PARP-1 has been revealed as a possible target in colorectal cancer, which is the second leading cause of cancer-related death worldwide. In this work, we screened a compound library to identify novel PARP inhibitors with low cytotoxicity and tested their efficacy in colorectal cancer cell models with and without defects in the DNA damage response. Furthermore, we evaluated whether the putative PARP inhibitors synergize with chemotherapeutic drugs used in the clinics to treat colorectal cancer patients. Using various experimental approaches, we were able to identify two promising molecules with potent PARP inhibition in colorectal cancer cells without causing cytotoxicity on their own. Moreover, the novel PARP inhibitors sensitized colorectal cancer cells to the anticancer drug irinotecan dependent on homologous recombination deficiency. Remarkably, the clinically approved PARP inhibitor olaparib displayed the strongest synergistic effects, but it was also cytotoxic as a single agent in wildtype colorectal cancer cells. The novel PARP inhibitors might, therefore, be useful for a combination therapy with irinotecan to avoid overlapping toxicity on healthy tissue such as bone marrow, which warrants further preclinical studies.

**Abstract:**

The DNA repair protein PARP-1 emerged as a valuable target in the treatment of tumor entities with deficiencies of *BRCA1/2*, such as breast cancer. More recently, the application of PARP inhibitors (PARPi) such as olaparib has been expanded to other cancer entities including colorectal cancer (CRC). We previously demonstrated that PARP-1 is overexpressed in human CRC and promotes CRC progression in a mouse model. However, acquired resistance to PARPi and cytotoxicity-mediated adverse effects limit their clinical applicability. Here, we detailed the role of PARP-1 as a therapeutic target in CRC and studied the efficacy of novel PARPi compounds in wildtype (WT) and DNA repair-deficient CRC cell lines together with the chemotherapeutics irinotecan (IT), 5-fluorouracil (5-FU), and oxaliplatin (OXA). Based on the ComPlat molecule archive, we identified novel PARPi candidates by molecular docking experiments in silico, which were then confirmed by in vitro PARP activity measurements. Two promising candidates (X17613 and X17618) also showed potent PARP-1 inhibition in a CRC cell-based assay. In contrast to olaparib, the PARPi candidates caused no PARP-1 trapping and, consistently, were not or only weakly cytotoxic in WT CRC cells and their BRCA2- or ATR-deficient counterparts. Importantly, both PARPi candidates did not affect the viability of nonmalignant human colonic epithelial cells. While both olaparib and veliparib increased the sensitivity of WT CRC cells towards IT, no synergism was observed for X17613 and X17618. Finally, we provided evidence that all PARPi (olaparib > veliparib > X17613 > X17618) synergize with chemotherapeutic drugs (IT > OXA) in a BRCA2-dependent manner in CRC cells, whereas ATR deficiency had only a minor impact. Collectively, our study identified novel lead structures with potent PARP-1 inhibitory activity in CRC cells but low cytotoxicity due to the lack of PARP-1 trapping, which synergized with IT in homologous recombination deficiency.

## 1. Introduction

Despite ongoing progress in the development of new approaches for CRC therapy, the five-year survival rate is still low, making it the second leading cause of cancer-related death worldwide [1]. An increasing incidence was observed primarily among younger age groups, which was attributed to changing lifestyle, medication, and environmental factors [2]. Current treatment of advanced CRC is based on surgery and chemotherapy with the DNA damage-inducing drugs irinotecan (IT), oxaliplatin (OXA) and 5-fluorouracil (5-FU), which are combined in several chemotherapy regimens [3]. Both the development of resistance during prolonged application and systemic toxicity limit the efficacy of currently used therapeutics [4].

Colorectal carcinogenesis is associated with (epi)genetic alterations of DNA repair. In hereditary CRC, mutations of DNA mismatch repair (MMR) genes such as *MLH1* and *MSH2* give rise to microsatellite instability (MSI) [5]. The MMR gene *MLH1* can also be epigenetically inactivated in sporadic CRC together with other genes, which is referred to as a CpG island methylator phenotype (CIMP) and results in MSI as well [5]. In contrast, the vast majority of sporadic CRC cases (up to 90%) arise through the chromosomal instability pathway (CIN), which is characterized by microsatellite stability (MSS) [6]. Furthermore, sporadic CRC formation is frequently accompanied by epigenetic inactivation of *MGMT* involved in the repair of DNA alkylation damage [7]. Mutations of *BRCA2* required for homologous recombination (HR)-mediated DNA repair occur rarely but were observed predominantly in young patients [8]. However, they have not been causally linked to an increased CRC susceptibility, unlike *BRCA1* mutations [9]. Interestingly, a more comprehensive study revealed that around 14% of all CRC cases exhibit HR deficiency (HRD) [10].

DNA damage induction represents the primary mechanism of anticancer drugs used in CRC treatment [11]. A fundamental component of the DNA damage response (DDR) is the enzyme poly (ADP-ribose) polymerase 1 (PARP-1) [12,13]. After binding to DNA strand breaks, PARP-1 is activated and catalyzes the post-translational formation of poly ADP-ribose (PAR) on various acceptor proteins, associated with DNA repair, histone modification, or cell cycle progression [13,14]. We found that PARP-1 is overexpressed in human CRC tissue, correlating with disease progression [15]. Using a CRC mouse model, we further demonstrated that PARP-1 protects against colorectal tumor induction, whereas it promoted colorectal tumor progression driven by intestinal inflammation [15]. These findings highlight the potential benefit arising from pharmacological PARP inhibition in CRC.

In recent years, several PARP inhibitors (PARPi) have been clinically approved for the treatment of ovarian and breast cancer [12,16]. The PARPi olaparib, rucaparib, and niraparib were shown to induce synthetic lethality in tumors deficient in BRCA1/2 [17]. The application of PARPi is currently expanded beyond BRCA1/2 deficiency to malignancies with other defects in HR and extensive testing may identify patient populations that benefit from PARPi treatment [18,19]. This includes defects of the apical DDR kinases ATM and ATR, as well as RAD51 involved in HR [20]. Mutations of *MRE11*, which is important for the detection of DNA double-strand breaks (DSBs), were reported to occur in CRC with MSI [21] and lead to higher cytotoxicity of PARPi in vitro [22] but not in patients receiving monotherapy [23]. Deficiency of ATM, which is observed in up to 10% of CRC cases, is accompanied by sensitivity towards olaparib, especially in the absence of wildtype (WT) p53 [24]. Although the anticancer effects of PARPi generally rely on mutations in DDR genes, multiple lines of evidence suggest a synergistic effect of PARPi and conventional chemotherapy also in tumors without genetic alterations of DNA repair [12,25]. Molecular susceptibilities beyond HRD have been identified in CRC [19]. Increased sensitivity towards the PARP inhibitor olaparib was found in patient-derived HROC278-Met cells containing a *BRAF* mutation [26]. Furthermore, *KRAS* mutant intrahepatic cholangiocarcinoma cells were shown to be highly sensitive to PARP inhibition [27], which might also hold true for *KRAS* mutated CRC. Despite these promising results from preclinical and clinical studies, the application of PARPi in cancer therapy is limited due to the development of PARPi resistance and adverse effects such as bone marrow toxicity, fatigue, and gastrointestinal toxicity [28,29].

In view of the emerging role of PARP-1 as a therapeutic target in CRC and the limitations observed for established PARPi, we aimed to identify PARPi with novel scaffolds and lower toxicity, in order to test their activity in MSI and MSS CRC cell lines without or with DDR defects. By in silico analysis, a set of 12 possible PARP inhibitors was identified and tested with a recombinant PARP-1 enzyme activity assay. The four most potent compounds were then used in an immunofluorescence-based PARP-1 activity assay applying human CRC cells. After identifying the two most potent inhibitors, their potential to cause PARP-1 trapping and their cytotoxicity was investigated in CRC cells proficient or deficient for PARP-1, BRCA2, and ATR and compared to human colonic epithelial cells (HCEC). Furthermore, the putative synergism with the chemotherapeutics IT, OXA, and 5-FU was investigated in CRC cell models with and without the DDR defects.

## 2. Material and Methods

### 2.1. Test Compounds

Synthesis of test compounds X17613, X17618, X17620, and X17621, and analysis by ESI mass spectrometry and NMR spectroscopy were conducted as described in SI material and methods. ^1^H and ^13^C-NMR spectra are shown in Supplementary Figures S1–S8. The test compounds X17608, X17610, X17611, X17616, X4739, X5157, X9563, and X12750 were kindly provided by ComPlat (KIT, Karlsruhe, Germany).

### 2.2. Molecular Docking

The virtual screening was calculated and analyzed using Schrödinger release 2020-4 (Schrödinger, LLC, New York, NY, USA, 2021). All protein structures (RCSB PDB: 4PJT [30], 7AAC, 7AAD [31]) were prepared using the Protein Preparation Wizard [32] by adding hydrogen atoms, assigning OPLS3e force field parameters [33], and replacing missing side chains or loops with Prime [34], followed by H-bond assignment optimization and restraint minimization. Before molecular docking, all compounds of the ComPlat library were prepared with LigPrep to predict possible protonation states and configurations. The hydrogen bonds to either G863 or S904 were set as constraints for Glide grid generation. Next, molecular docking with flexible ligands and rigid protein conformation was performed with Glide SP scoring [35]. Finally, the binding poses were visually inspected and re-scored with Glide XP to obtain the binding free energy values [36].

### 2.3. PARP Inhibitor Assay

Twelve compounds were screened regarding their ability to inhibit PARP-1 enzyme activity in vitro. Therefore, we utilized a chemiluminescence-based PARP-1 assay kit (BPS Bioscience, San Diego, CA, USA) and performed two independent experiments according to the manufacturer’s instructions. Briefly, non-transparent 96-well plates were coated with histones overnight. Substances were tested in duplicates, applying concentrations of 0.001, 0.01, 0.1, 1, 10, and 100 µM. Olaparib and veliparib in concentrations of 5 and 50 nM served as positive controls. After adding activated DNA and biotinylated NAD^+^, the ADP-ribosylation reaction was initiated by the addition of PARP-1 enzyme and incubated at RT for 1 h. Quantification of biotinylated PAR was conducted by using Streptavidin–HRP and subsequent ECL-based detection of chemiluminescence, applying a Spark^®^ multiplate reader (Tecan, Crailsheim, Germany). The lowest applied concentration was set to 100% and IC_50_ values were calculated by nonlinear regression using GraphPad Prism 9.0 Software (GraphPad Software Inc., Boston, MA, USA).

### 2.4. Cell Culture and Treatments

Genetically engineered HCT116 PARP-1^+/+^ and HCT116 PARP-1^−/−^ were generated in 2017 by CRISPR-based targeting as described elsewhere [15]. Wildtype HCT116 cells were obtained from the Core Cell Center (John Hopkins University, Baltimore, MD, USA) in 2012, while HCT116 BRCA2^−/−^ cells were kindly provided by Dr. Carlos Caldas in 2017 (University of Cambridge, Cambridge, UK) [37]. Parental DLD-1 ATR^+/+^ and DLD-1 ATR^s/s^ cells were generated by Dr. Fred Bunz (John Hopkins University, Baltimore, USA) [38] and obtained in 2018. Caco-2 cells were obtained from CLS Cell Lines Service (Eppelheim, Germany) in 2012. Non-transformed human colonic epithelial cells (HCEC; 1CT) were established by Dr. Jerry W. Shay (UT Southwestern Medical Center, Dallas, TX, USA) [39] and kindly provided in 2015. Cells lines were re-authenticated by p53, PARP-1, ATR, and BRCA2 immunoblotting, by their characteristic differential response to genotoxic agents and their typical cell morphology. HCT116, HCT116 BRCA2^−/−^, DLD-1 ATR^+/+^ and DLD-1 ATR^s/s^ cells were maintained in DMEM, whereas HCT116 PARP-1^+/+^ and HCT116 PARP-1^−/−^ cells were maintained in RPMI1640 containing 10% FCS and 1% penicillin/streptomycin in a humidified atmosphere at 37 °C and 5% CO_2_. Caco-2 cells were cultured in MEM with 10% FCS, 1% penicillin/streptomycin, and nonessential amino acids. HCECs were grown in a nitrogen incubator with reduced oxygen levels (7% O_2_) and 5% CO_2_ at 37 °C in DMEM GlutaMax/Medium 199 (4:1) with supplements as reported previously [40]. The media and supplements were obtained from PAN-Biotech (Aidenbach, Germany) and Thermo Fisher Scientific (Darmstadt, Germany). Cell culture was frequently tested for contamination with mycoplasma by PCR using the Venor^®^ GeM Classic kit (Minerva Biolabs, Berlin, Germany) and immunofluorescence microscopy with nuclear staining. The PARP inhibitors olaparib and veliparib were from MedChemExpress and bought at Hycultec (Beutelsbach, Germany). The PARP inhibitors as well as the test compounds were dissolved in DMSO as 10 mM stock solution and used in cell culture experiments in a final concentration range from 0 to 50 µM. DMSO, in a concentration equivalent to the highest inhibitor concentration used, served as a negative control (0 µM). Cells were exposed for 1 h for the quantification of PARP-1 trapping or 2 h for assessing the inhibitory potential by confocal fluorescence microscopy. PARP activity was either induced by methyl methanesulfonate (MMS) cotreatment (1 mM) for 1 h (PARP trapping) or by treatment with H_2_O_2_ (1 mM) for 5 min subsequent to inhibitor incubation. The chemotherapeutic drugs 5-FU, IT, and OXA (all from MedChemExpress and purchased at Hycultec, Beutelsbach, Germany) were dissolved in water or DMSO as 75 mM, 34 mM, and 10 mM stock solution, respectively, and used as indicated. If the PARPi treatment was conducted in combination with cytostatic drugs for 24 h (γH2AX Western blot analysis) or 72 h (cytotoxicity testing), the PARPi was added 2 h prior.

### 2.5. Cytotoxicity Testing of PARP Inhibitors

To assess the cytotoxicity of newly developed PARP inhibitors, cells were cultivated in translucent 96-well plates overnight and treated with PARP inhibitors or cytostatic drugs for 72 h. For the assessment of synergistic activity, cells were pretreated with the inhibitors for 2 h, before the inhibitor or cytostatic drug was added. Cell viability was assessed by the resazurin reduction assay (RRA) as described previously [41], and fluorescence was measured using a Spark^®^ Multi-well reader (Tecan, Crailsheim, Germany). IC_50_ values were calculated using GraphPad Prism 9.0 software (GraphPad Software Inc., Boston, MA, USA). To this end, concentrations were transformed into the log scale, plotted against the cell viability and the curve was fitted by nonlinear regression with variable slope, providing the IC_50_ values.

### 2.6. PAR Immunofluorescence Analysis

The activity of potential PARP inhibitors was tested in a cell model using immunofluorescence-based detection of PAR essentially as reported previously [15]. HCT116 cells were seeded in Ibidi 12-well chamber slides and cultivated until they reached 70% confluence. Induction of PARylation was achieved by adding 1 mM H_2_O_2_ in PBS/1 mM MgCl_2_ for 5 min at 37 °C. Cells were washed with PBS/1 mM MgCl_2_ and fixed by adding 4% PFA at RT. After 20 min, cells were washed with PBS/100 mM glycine for 1 min and permeabilized with PBS/0.3% Triton X-100 for 3 min. Blocking of the cells was conducted by adding PBS/0.05% Tween containing 5% powdered milk for 1 h at RT. Immunofluorescence staining of PAR was performed by either adding a PAR antibody clone 10H or pan-ADP-ribose binding reagent (Sigma-Aldrich, Saint Louis, MO, USA) diluted 1:300 in PBS/0.05% Tween containing 5% powdered milk overnight at 4 °C. Cells were washed and an Alexa488-coupled secondary antibody was added for 1 h at RT. The slides were mounted with VectaShield containing DAPI and analyzed by confocal microscopy using a Zeiss Axio Observer 7 microscope (Oberkochen, Germany) equipped with a 63× oil objective (Plan-Apochromat 63×/1.40 DIC M27) and a LSM900 confocal laser scanner (Zeiss, Oberkochen, Germany). Images were analyzed using Zeiss Zen software version 3.4 and ImageJ v1.53t (NIH, Bethesda, MD, USA). Briefly, nuclei were identified based on the DAPI signal and marked as regions of interest (ROI). Subsequently, the mean PAR signal in the identified ROI was quantified in each image and exported for further analysis. For the individual experiments, the mean PAR intensity was averaged for each treatment group and the control was set to 100%. Only images with a minimum of 4 cells were included in the analysis and at least 5 images were assessed per treatment group. The experiments were conducted in at least 3 biological replicates. To derive IC_50_ concentrations, relative PAR levels of the experiments were transferred to GraphPad Prism 9.0 Software (GraphPad Software Inc., Boston, MA, USA) for a nonlinear regression analysis applying a three-parameter model.

### 2.7. Chromatin Retention Assay and Western Blot Analysis

Quantification of chromatin-trapped PARP-1 was performed as described elsewhere [42]. Briefly, HCT116 cells were harvested, and pellets were lysed in buffer containing 150 mM KCl, 2.5 mM MgCl_2_, 50 mM HEPES pH 7.8, 5 mM EDTA pH 8, 3 mM dithiothreitol (DTT), 10% glycerol, 0.5% Triton X-100, and freshly added protease inhibitor cocktail (Roche) for 15 min on ice. The chromatin-bound protein fraction was isolated by centrifugation at 4 °C for 15 min and 16.000× *g*. After transferring the soluble fraction, the chromatin-containing pellet was washed twice in lysis buffer and sonicated for 3 min. The soluble and chromatin fractions were mixed with self-made 5× Laemmli buffer (200 mM TRIS pH 6.8, 40% glycerol, 8% SDS, 4 % ß-mercaptoethanol, 0.08% Bromphenol Blue) and incubated at 95 °C for 10 min. Samples were then separated by SDS-PAGE followed by Western blot analysis essentially as described previously [43]. For the analysis of γH2AX, cells were directly harvested in 1× Laemmli buffer, incubated at 95 °C for 5 min, and subjected to SDS-PAGE and subsequent Western blot analysis [44]. The following primary antibodies were used: anti-PARP-1 (#GTX112864, Genetex, Irvine, CA, USA), anti-Histone H3 (#GTX122148, Genetex), anti-heat shock protein (Hsp90) α/ß (#sc-13119, Santa Cruz Biotechnology, Heidelberg, Germany), anti-γ-H2AX (#ab81299, Abcam, Cambridge, UK). The following secondary antibodies were used: anti-rabbit IgG-HRP (#7074, Cell Signaling Technology, Danvers, MA, USA) and m-IgGκ binding protein-HRP (#sc-516102, Santa Cruz Biotechnology). The proteins of interest were detected using a c300 chemiluminescence imager (Azure Biosystems, Dublin, CA, USA). Densitometric image analysis was conducted by applying the software AzureSpot version 2.0.062 (Azure Biosystems, USA). The signal intensity (e.g., PARP-1 or γH2AX) of each lane was quantified and subsequently normalized to the respective loading control as indicated in the figure legends, which was analyzed in each experiment. Finally, the normalized signal intensity of each treatment group was expressed relative to the negative control and transferred to GraphPad Prism 9.0 Software (GraphPad Software Inc., Boston, MA, USA) for statistical analysis (see Section 2.8).

### 2.8. Statistics

Experiments were performed independently at least three times, except otherwise stated. Results from representative experiments are depicted. Values underwent Grubbs’ test to exclude outliers and are displayed as mean +/− standard error of the mean (SEM) using the GraphPad Prism 9.0 Software (GraphPad Software Inc., Boston, MA, USA). Statistical analysis was performed using a two-sided Student’s *t*-test and statistical significance was defined as *p* < 0.05.

## 3. Results

### 3.1. Identification of Putative PARPi Using Molecular Docking Studies

**Figure 1:**
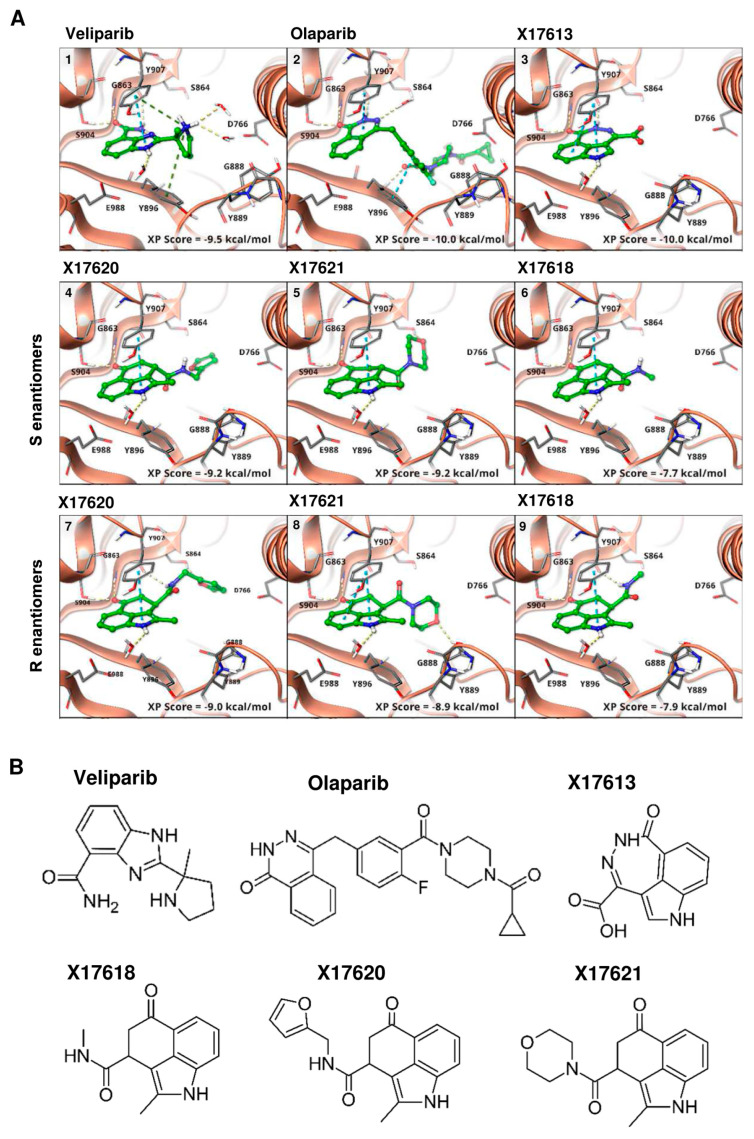
(**A**) Binding modes of veliparib (**1**), PDB: 7AAC), olaparib ((**2**), PDB: 7AAD), and selected compounds from virtual screening ((**3**–**9**), PDB: 4PJT) to PARP-1. The binding to either G863 or S904, as also found for veliparib, was used as a constraint in docking. All active compounds are able to form this bond and adopt a similar binding mode. Through the indole NH, there is an interaction with E988 by a bridging water molecule. No preference between the binding modes of the S- (**4**–**6**) or R-enantiomers (**7**–**9**) is observed, while the scoring values also differ only slightly. (**B**) Chemical structure of the most active compounds X17613, X17618, X17620, and X17621, according to in vitro screening and the two established PARPi veliparib and olaparib.

**Figure A1:**
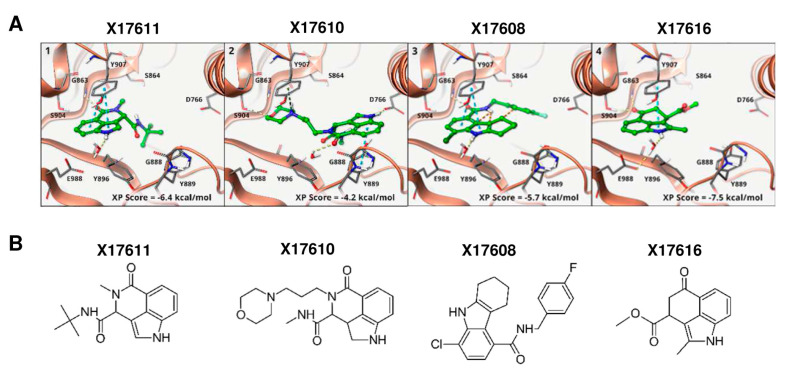
(**A**) Binding modes of selected compounds from virtual screening ((**1**–**4**), PDB: 4PJT) to PARP-1. The binding to either G863 or S904 was used as a constraint in docking. All active compounds are able to form this bond and adopt a similar binding mode (compare Figure 1A). The three compounds X17611, X17610, and X17608 (**1**–**3**) serve as a verification of the binding mode since the constraints can only be fulfilled with significant losses in the binding free energy due to unfavorable inter- and intramolecular interactions. X17616 (**4**) is similar to X17618 (Figure 1A), but cannot form a potential hydrogen bond due to the lack of the amide function. (**B**) Chemical structure of the compounds X17611, X17610, X17608 and X17616.

We devised a focused library of 3,4-bifunctionalized and -bridged indoles that fill an underrepresented chemical space within the vast number of reported indole derivatives as privileged scaffolds in drug discovery. From in silico screening of this focused in-house library within the whole ComPlat archive with more than 18,000 molecules, four compounds were identified with similar docking scores and binding poses compared to the well-established PARP inhibitors veliparib and olaparib (Figure 1(A1,A2)). In silico analysis revealed that the most active compound, X17613 with the carboxylic acid hydrazide motif, forms key interactions with G863, S904, and Y907 (Figure 1(A3)), while hydrogen bonding to the carbonyl oxygen atom of G863 is not mandatory. The derivatives X17618, X17620, and X17621, nevertheless, suggested PARP-1 binding affinity in silico, albeit lower, while alkylation of the amide of, e.g., X17611 would lead to a clash with the backbone carbonyl oxygen atom of G863 (Appendix A, Figure A1(A1)). Furthermore, a binding mode allowing hydrogen bonding to G863 or S904 could be identified for almost no compound substituted at this position. Hence, for compound X17610, we obtained a completely different binding mode, where the indole NH could interact with D766, and the hydrogen bond to S904 is formed via the morpholine oxygen atom (Appendix A, Figure A1(A2)). Therefore, we tested this compound despite a shallow scoring value to evaluate the possibility of other binding modes. Although X17608 is structurally similar to veliparib, the docking score is significantly reduced due to an intramolecular clash in a similar binding mode (Appendix A, Figure A1(A3)). All chiral compounds were tested as racemates, and no favored binding modes for the individual enantiomers could be identified by molecular docking (Figure 1(A4–A9)). To sum up, in silico studies revealed X17613 as the compound with the highest PARP-1 binding affinity, followed by the derivatives X17618, X17620, and X17621 (for chemical structures see Figure 1B and Appendix A, Figure A1B).

### 3.2. Activity Screening of PARP Inhibitors

**Figure 2:**
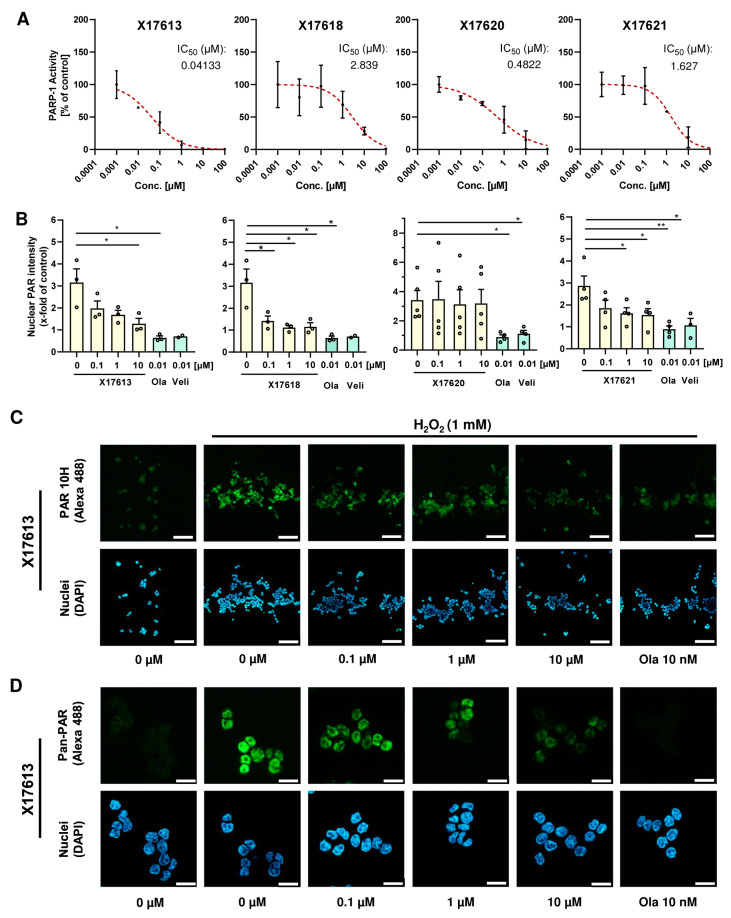
(**A**) Concentration–response curves of four potential PARP-1 inhibitors with the highest activity in the PARP-1 screening assay kit. All concentrations were tested in duplicates. IC_50_ values were derived using a nonlinear regression model in GraphPad Prism 9 (*n* = 2). (**B**) Investigation of PARP inhibition by X17613, X17618, X17620, and X17621 in HCT116 cells. Cells were challenged with 1 mM H_2_O_2_ for 5 min and pretreated or not with the indicated compounds for 2 h. PAR synthesis was identified by confocal IF microscopy using the PAR 10H antibody. The signal intensity of five images per concentration was evaluated by ImageJ (*n* ≥ 3). (**C**) Representative confocal microscopy images at 100× magnification after PAR staining in HCT116 cells treated with the indicated concentrations of X17613 for 2 h with or without subsequent PARP activation by H_2_O_2_ treatment for 5 min. Scale bar: 100 µm. (**D**) Confocal microscopy images at 630× magnification after pan-PAR staining in HCT116 cells treated according to (**C**). Scale bar: 20 µm. Data are presented as mean +/− SEM. * *p* < 0.01, ** *p* < 0.01; *t*-test.

**Figure A2:**
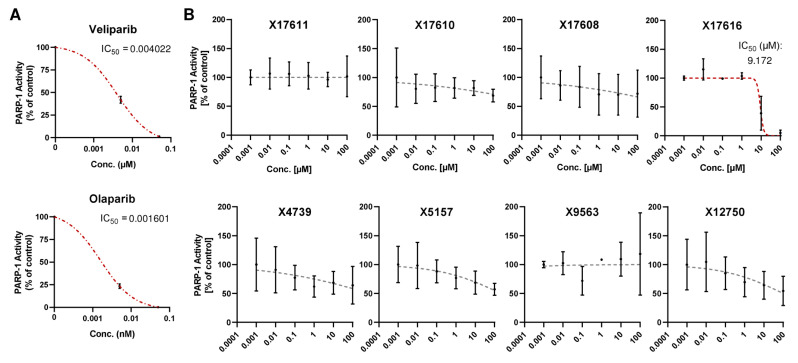
(**A**,**B**) Concentration response curve of established PARP inhibitors veliparib and olaparib (**A**) and eight potential PARP inhibitors (**B**) with low or no activity in the PARP-1 screening assay kit. All concentrations were tested in duplicates. IC_50_ values were derived using a nonlinear regression model in GraphPad Prism 9 (*n* = 2).

After the molecular docking studies, twelve selected compounds were tested with regard to their potential to inhibit PARP-1 in a chemiluminescent PARP-1 screening assay kit. Five test compounds led to a concentration-dependent decrease in PARylation catalyzed by PARP-1, whereas the other seven compounds had no or only little effect on PARP-1 activity (Figure 2A and Appendix A, Figure A2B). The calculated IC_50_ values varied between 41 nM for the most potent compound X17613 and 9.2 µM for the compound X17616 with the lowest inhibitory potency. In general, these IC_50_ values are higher than the inhibitory activity observed for the positive controls olaparib and veliparib (Appendix A, Figure A2A), which are in the low nM range (1.6 nM and 4 nM, respectively). Applied in a concentration of 5 nM, olaparib and veliparib reduced PARP-1 activity to approximately 25% and 50% of the negative control, respectively. The most potent compounds X17613, X17618, X17620, and X17621 were selected for further testing in a cell-based screening of PARP-1 activity (see below). Overall, the determined IC_50_ values of the tested compounds correlated well with the Glide XP scoring values of the determined docking poses from virtual screening (Figure 1A, and Appendix A, Figure A1A).

### 3.3. Effect of Selected Test Compounds on PAR Formation in HCT116 Cells

**Figure A3:**
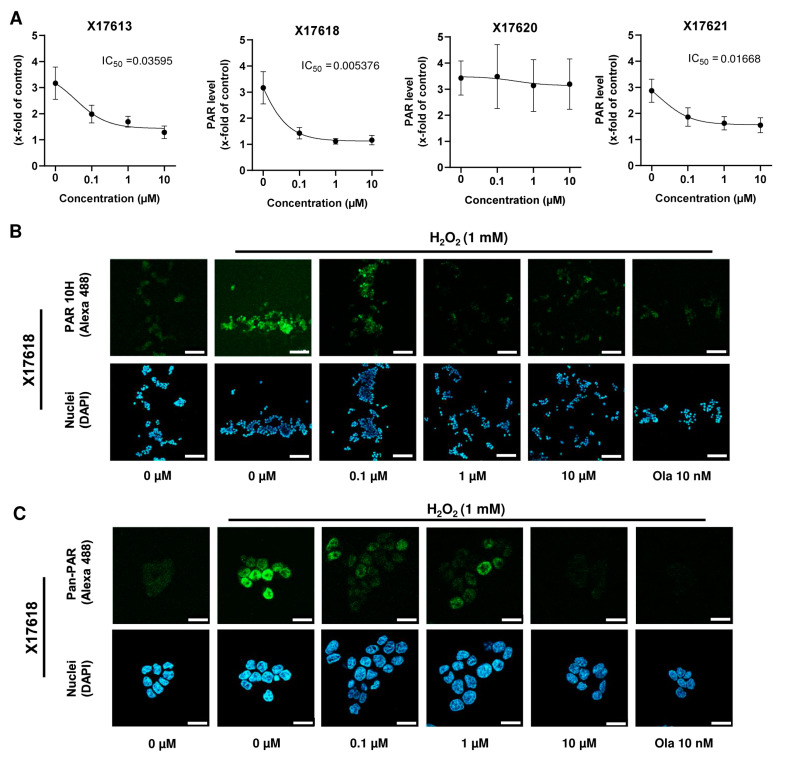
(**A**) Concentration-response curves of 4 potential PARP-1 inhibitors (X17613, X17618, X17620 and X17621) assessed in HCT116 cells as described in Figure 2B (*n* = 3). IC_50_ values were derived using a nonlinear regression model in GraphPad Prism 9. (**B**) Representative confocal microscopy images at 100× magnification after PAR staining in HCT116 cells treated with the indicated concentrations of X17618 for 2 h with or without subsequent PARP induction by H_2_O_2_ treatment for 5 min. Scale bar: 100 µm. (**C**) Confocal microscopy images at 630× magnification after pan-PAR staining in HCT116 treated as described in B. Scale bar: 20 µm.

Further assessment of the PARPi activity of the four most potent compounds X17613, X17618, X17620, and X17621 was conducted in HCT116 CRC cells (Figure 2B). The inhibitory potential of the compounds on H_2_O_2_-induced PAR formation was measured by PAR antibody staining and subsequent analysis by confocal fluorescence microscopy (Figure 2C and Figure A3A). A significant decrease in the PAR signal was observed for the inhibitors X17613, X17618, and X17621 already at a concentration of 100 nM. In contrast to that, the compound X17620 showed no effect on PAR formation in the cell-based assay even at a concentration of 10 µM (Figure 2B). Unlike the inhibitors X17613 and X17618, the compound X17621 did not entirely inhibit PAR formation at the highest concentration. As a positive control, 10 nM olaparib was included, which completely blocked PAR formation (Figure 2B,C). Since the applied 10H PAR-Antibody detects PAR in a chain length-dependent manner with preferential binding to PAR polymers consisting of more than 20 monomers [45], we also conducted immunostaining with the pan-ADP-ribose binding reagent using the same protocol. Fluorescence microscopy revealed comparable results to the 10H clone PAR antibody (Figure 2D and Appendix A, Figure A3B). Taken together, the three compounds X17613, X17618, and X17621 showed a similar potency for cellular PARP inhibition following DNA damage induction by H_2_O_2_ with IC_50_ values between 5 and 35 nM (Figure 2B and Appendix A, Figure A3C). In contrast to the PARPi studies with recombinant PARP-1 enzyme, X17620 was not active in the cell model and, thus, was excluded from further testing.

### 3.4. PARP-1 Trapping and Cytotoxicity of PARP Inhibitors

**Figure 3:**
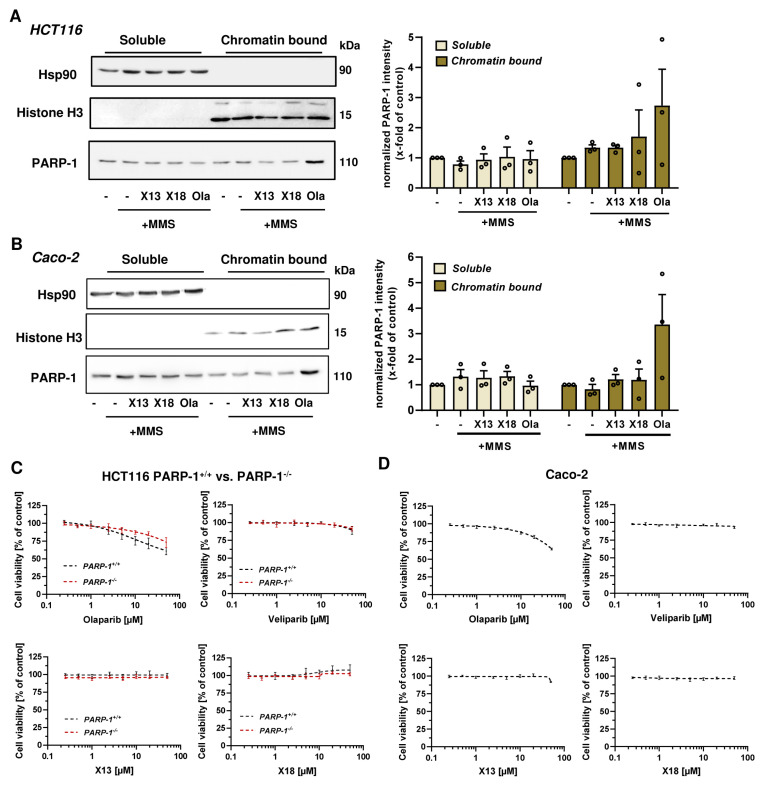
(**A**,**B**) Analysis of PARP-1 trapping in HCT116 and Caco-2 cells. Immunoblot detection of PARP-1 after pre-treatment with X17613, X17618, and olaparib followed by MMS exposure for 1 h and chromatin isolation. The cytosolic marker Hsp90 and the chromatin marker Histone H3 served as respective loading controls. Representative Western blot images and densitometric evaluation are shown (*n* = 3). Data are shown as mean + SEM. (**C**) Cell viability determined by the resazurin reduction assay (RRA) in HCT116 PARP-1^−/−^ and HCT116 PARP-1^+/+^ cells after PARPi treatment for 72 h. A nonlinear regression curve fit was conducted using GraphPad Prism 9 (*n* ≥ 3). (**D**) Viability in Caco-2 cells after exposure to PARPi as indicated. (*n* = 3). All data are shown as mean +/− SEM.

**Figure A4:**
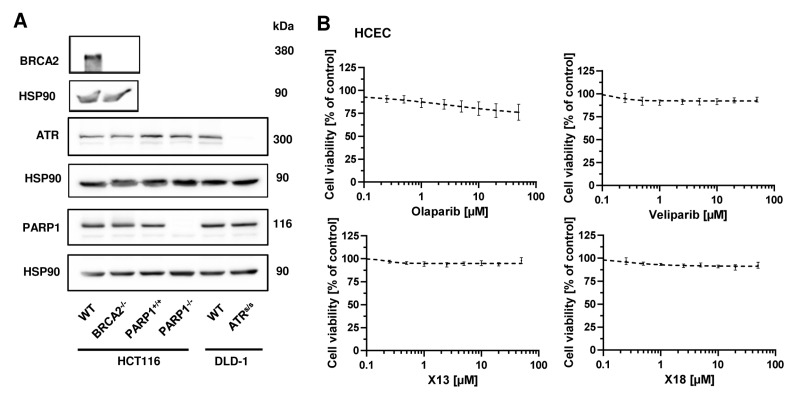
(**A**) Western Blot analysis of PARP-1, BRCA2 and ATR expression in HCT116 WT, HCT116 BRCA2^−/−^, HCT116 PARP-1^+/+^, HCT116 PARP-1^−/−^, DLD-1 WT and DLD-1 ATR^−/−^ cells. HSP90 served as loading control. (**B**) Toxicity of PARPi in human colonic epithelial cells (HCEC). Cells were incubated with PARPi for 72 h and viability was assessed using the RRA. Nonlinear regression curve fit was conducted using GraphPad Prism 9 (*n* ≥ 3).

In the next step, we focused on X17613 and X17618 with the highest in vitro and in cellulo PARPi activity and analyzed their potential to cause PARP-1 trapping. To this end, chromatin isolation was performed in MSI HCT116 cells after PARP activation by exposure to the alkylating agent MMS for 1 h with or without an inhibitor. The results showed that only olaparib caused a substantial increase in chromatin-bound PARP-1 (Figure 3A). No enrichment of PARP-1 in the chromatin fraction was observed after treatment with X17613 or X17618, indicating that PARP-1 trapping is not induced by these inhibitors at the used concentrations. The same set of experiments was repeated in MSS Caco-2 cells, revealing comparable results. Olaparib caused strong PARP-1 trapping, whereas no effects were detected after treatment with X17613 and X17618 (Figure 3B). Since the ability of PARPi to trap PARP-1 is associated with their cytotoxic potential and side effects in vivo, we assessed the viability in HCT116 cells depending on PARP-1. Prior to that experiment, Western blot analysis was used to validate the lack of PARP-1 protein expression in HCT116 PARP-1^−/−^ cells, while HCT116 PARP-1^+/+^ control cells displayed PARP-1 expression as expected (Appendix A, Figure A4A). Neither X17613 nor X17618 decreased cell viability in HCT116 cells, irrespective of the PARP-1 status (Figure 3C). Veliparib displayed only mild cytotoxicity at the highest test concentration. In contrast to that, olaparib decreased viability in HCT116 cells in a concentration-dependent manner, which was much more pronounced in cells expressing PARP-1 (Figure 3C). Moreover, the effects of the established and novel PARPi on the viability of MSS Caco-2 cells were determined. X17618 had no impact on cell viability at all, while veliparib and X17613 showed little cytotoxicity at the highest test concentration (Figure 3D). In line with the findings in HCT116 cells, olaparib treatment resulted in a concentration-dependent reduction in Caco-2 viability (Figure 3D). Finally, we evaluated the cytotoxic potential of PARPi in human colonic epithelial cells (HCEC), which were established from human healthy colon biopsies. While olaparib caused a moderate decline in viability at the highest concentration, the other PARPi (veliparib, X17613, and X17618) had no effect on HCEC (Appendix A, Figure A4B) Taken together, the novel PARPi X17613 and X17618 did not trap PARP-1 and, similar to veliparib, induced no cytotoxicity in two different CRC cell models (MSI and MSS) and HCEC (Table 1). Olaparib in turn caused substantial PARP-1 trapping and was cytotoxic in CRC cells in a PARP-1-dependent manner.

### 3.5. Cytotoxicity of PARPi Depending on the Cellular BRCA2 and ATR Status

**Figure 4:**
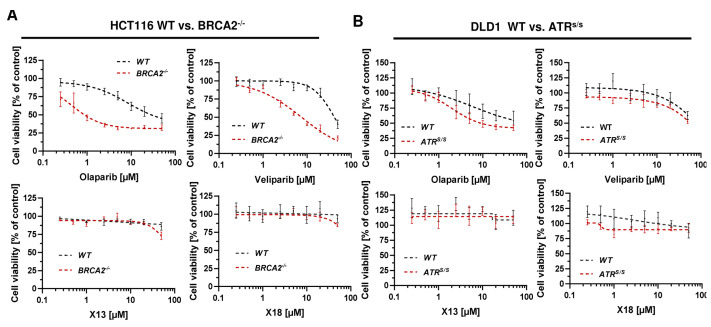
(**A**) Toxicity of PARPi in HCT116 cells depending on BRCA2 status. HCT116 WT and HCT116 BRCA2^−/−^ cells were incubated with PARPi for 72 h and viability was assessed using the resazurin reduction assay (RRA). Nonlinear regression curve fit was conducted using GraphPad Prism 9 (*n* ≥ 3). (**B**) Toxicity of PARPi in DLD-1 cells depending on ATR status. DLD-1 WT and DLD-1 ATR^s/s^ cells were incubated with PARPi for 72 h and viability was assessed using the RRA. Nonlinear regression curve fit was conducted using GraphPad Prism 9 (*n* ≥ 3). Data are depicted as mean +/− SEM.

To investigate the impact of the cellular DDR on the cytotoxicity of PARP inhibitors, we used genetically engineered CRC cell models proficient or deficient for *BRCA2* and *ATR*, respectively. Cell models were re-authenticated using Western blot analysis, which confirmed a lack of BRCA2 and ATR protein expression in the respective knockout model (Appendix A, Figure A4). Cytotoxicity testing revealed no impact of X17613 and X17618 on viability in HCT116 WT cells, whereas both compounds decreased viability in HCT116 BRCA2^−/−^ cells by about 25% at the highest test concentration of 50 µM (Figure 4A). High cytotoxicity in the low micromolar concentration range was observed in HCT116 BRCA2^−/−^ cells incubated with olaparib or veliparib. The determined IC_50_ values in BRCA2-deficient HCT116 cells were 18-times lower for olaparib and 5-times lower for veliparib in comparison to HCT116 WT cells (Table 1). Further experiments in DLD-1 WT and DLD-1 ATR^s/s^ cells showed no effects of X17613 and X17618, irrespective of the ATR status (Figure 4B). Veliparib caused a concentration-dependent decrease in viability in both cell models, but concentration-response data did not allow for deriving IC_50_ values. Olaparib showed the strongest cytotoxic effects of all tested PARPi, which was affected by the ATR status. DLD-1 ATR^s/s^ cells displayed a 3-fold higher sensitivity for olaparib than DLD-1 WT cells, as revealed by the respective IC_50_ values (Table 1). In summary, BRCA2 deficiency potentiated the sensitivity of CRC cells towards all tested PARPi (Olaparib » veliparib > X17613 ≈ X17618), while ATR deficiency only increased sensitivity towards olaparib.

### 3.6. Combination of PARPi and Clinically Relevant Chemotherapeutic Drugs in CRC Cells

**Figure A5:**
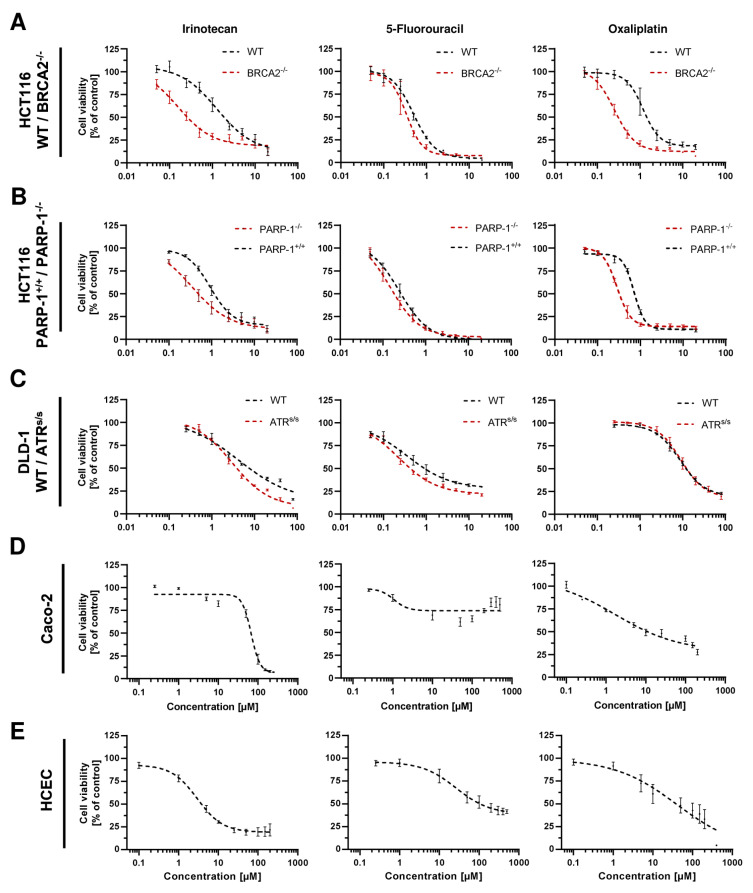
Cell viability of (**A**) HCT116 WT and HCT116 BRCA2^−/−^, (**B**) HCT116 PARP-1^+/+^ and HCT116 PARP-1^−/−^, (**C**) DLD-1 WT and DLD-1 ATR^s/s^, (**D**) Caco-2 cells and (**E**) HCEC after monotreatment with cytostatic drugs IT, 5-FU and OXA for 72 h. Nonlinear regression curve fit was conducted using GraphPad Prism 9 (*n* ≥ 3).

To investigate a putative synergism with anticancer drugs, IC_50_ values of IT, 5-FU, and OXA were first determined in all cell models (Appendix A, Figure A5). In HCT116 cells, the toxicity of IT and OXA were dependent on the molecular subtype. In comparison to HCT116 WT cells, HCT116 BRCA2^−/−^ was revealed to be more sensitive with an IC_50_ value 8-times lower for IT and 5-times lower for OXA, while no such difference was observed for 5-FU (Appendix A, Figure A5A and Table 1). The PARP-1 status also impacted the sensitivity of HCT116 cells to IT and OXA with 2.5–3-fold higher sensitivity in PARP-1^−/−^ cells, while the cytotoxicity of 5-FU was less affected (Appendix A, Figure A5B and Table 1). Experiments in DLD-1 cells showed little influence of ATR on cytotoxicity, as reflected by a 1.4-times lower IC_50_ value for IT and a 1.6-times lower IC_50_ value for 5-FU in DLD-1 ATR^s/s^ cells (Appendix A, Figure A5C and Table 1). The cytotoxic effects of OXA were independent of ATR. In Caco-2 cells, the cytotoxicity of the anticancer drugs was generally lower than in HCT116 or DLD-1 cells (Figure A5D and Table 1). Furthermore, nonmalignant HCEC were tested, which displayed similar sensitivity for IT but lower cytotoxicity for 5-FU and OXA than the CRC cell lines (Appendix A, Figure A5E).

**Figure 5:**
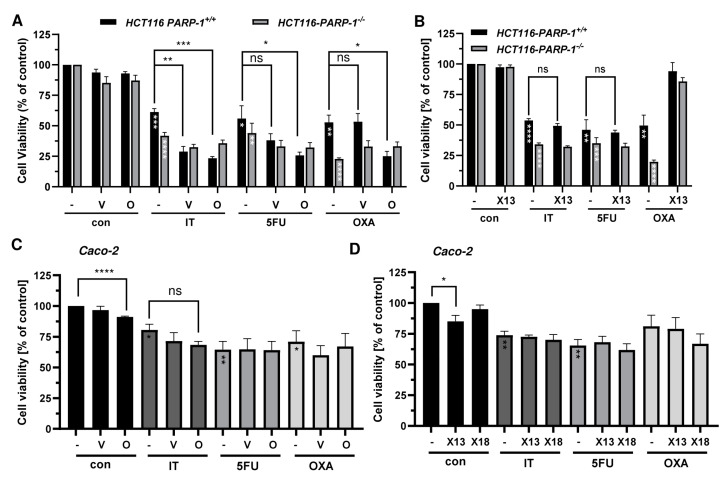
(**A**) Viability in HCT116 PARP-1^−/−^ and HCT116 PARP-1^+/+^ cells after treatment with PARPi olaparib or veliparib in combination with chemotherapeutic drugs irinotecan (IT, 0.5 µM), 5-fluorouracil (5-FU, 0.25 µM), and oxaliplatin (OXA, 0.5 µM) for 72 h (*n* ≥ 3). (**B**) Viability in HCT116 PARP-1^−/−^ and HCT116 PARP-1^+/+^ cells after treatment with PARPi X17613 in combination with chemotherapeutic drugs for 72 h. Data (*n* ≥ 3) are given as mean +/− SEM. (**C**,**D**) Viability in Caco-2 cells after treatment with PARPi in combination with chemotherapeutic drugs irinotecan (IT, 10 µM), 5-fluorouracil (5-FU, 5 µM), and oxaliplatin (OXA, 1 µM). Data (*n* = 3) are shown as mean +/− SEM. ns: *p* > 0.05, * *p* < 0.01, ** *p* < 0.01, *** *p* < 0.001, **** *p* < 0.0001; *t*-test.

**Figure A6:**
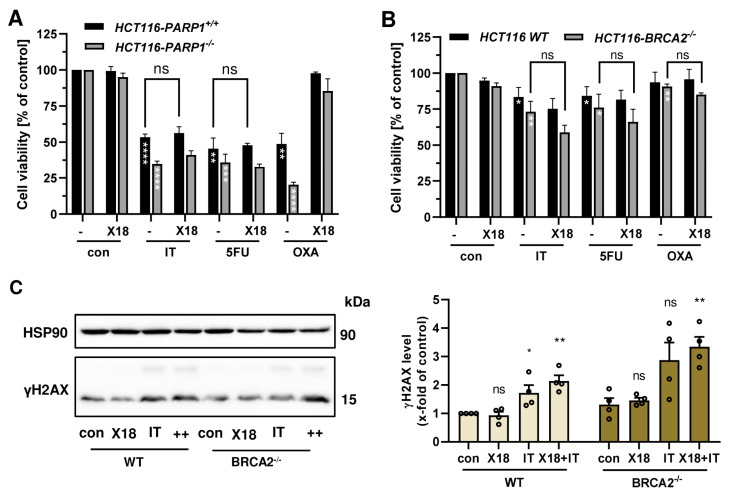
(**A**) Viability of HCT116 PARP-1^−/−^ and HCT116 PARP-1^+/+^ cells after treatment with PARPi X17618 in combination with chemotherapeutic drugs irinotecan (IT, 0.5 µM), 5-fluorouracil (5-FU, 0.25 µM) and oxaliplatin (OXA, 0.5 µM) for 72 h (*n* ≥ 3) (**B**) Viability of HCT116 WT and HCT116 BRCA2^−/−^ cells after treatment with PARPi X17618 in combination with chemotherapeutic drugs irinotecan (IT, 0.25 µM), 5-fluorouracil (5-FU, 0.1 µM) and oxaliplatin (OXA, 0.25 µM) for 72 h (*n* ≥ 3). (**C**) γH2AX formation in HCT116 WT and BRCA2^−/−^ cells after treatment with X17618 (50 µM), IT (0.25 µM) or a combination of both for 24 h. Representative Western blot images and densitometric evaluation are shown (*n* = 4). All data are presented as mean +/− SEM. ns: *p* > 0.05, * *p* < 0.01, ** *p* < 0.01, *** *p* < 0.001, **** *p* < 0.0001; *t*-test.

Subsequently, combined treatment of PARPi and DNA damage-inducing anticancer drugs was performed. We observed a significant decrease in cell viability compared to mono-treatment with cytostatics for olaparib and veliparib in HCT116 PARP-1^+/+^, whereas in HCT116 PARP1^−/−^ olaparib and veliparib did not sensitize to the anticancer drugs (Figure 5A). The novel compounds X17613 and X17618 did not affect the cytotoxicity of the anticancer drugs (Figure 5B and Appendix A, Figure A6A). In Caco-2, both olaparib and veliparib moderately increased the cytotoxicity of IT, which was, however, not statistically significant (Figure 5C).

No effects were observed for the combination treatment with the cytostatic drugs 5-FU and OXA. In line with the findings in HCT116 cells, no sensitization towards the anticancer drugs was detected upon incubation with X17613 and X17618 (Figure 5D). Collectively, these results showed an increased sensitivity of CRC cells (HCT116 > Caco-2) towards IT after treatment with olaparib and veliparib, while X17613 and X17618 had no synergistic effect. Furthermore, our data highlighted the relevance of PARP-1 expression for the cytotoxicity of both IT and OXA.

### 3.7. Impact of BRCA2 and ATR on the Potential Synergism of PARPi and Chemotherapeutics

**Figure 6:**
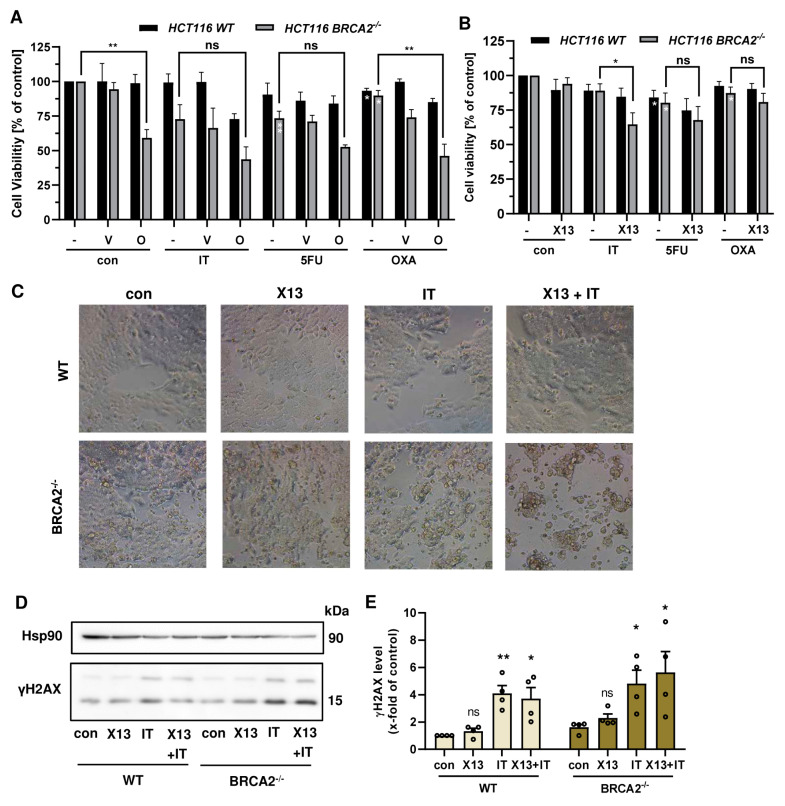
(**A**) Viability in HCT116 WT and HCT116 BRCA2^−/−^ cells after treatment with PARPi olaparib or veliparib in combination with chemotherapeutic drugs (IT, 0.25 µM), 5-fluorouracil (5-FU, 0.1 µM), and oxaliplatin (OXA, 0.25 µM) for 72 h (*n* ≥ 3). (**B**) Viability in HCT116 WT and HCT116 BRCA2^−/−^ cells after treatment with PARPi X17613 in combination with chemotherapeutic drugs (IT, 0.25 µM), 5-fluorouracil (5-FU, 0.1 µM), and oxaliplatin (OXA, 0.25 µM) for 72 h (*n* ≥ 3). (**C**) Representative brightfield microscopic images at 20X magnification of HCT116 WT and HCT116 BRCA2^−/−^ cells after treatment with X17613 (50 µM), IT (0.25 µM), or a combination of both for 24 h. (**D**,**E**) γH2AX formation in HCT116 WT and BRCA2^−/−^ cells after treatment as described in (**C**). Representative Western blot images and densitometric evaluation are shown (*n* = 4). Hsp90 served as loading control. All data are given as mean + SEM. ns: *p* > 0.05, * *p* < 0.01, ** *p* < 0.01; *t*-test.

Finally, we studied how the DDR status (BRCA2 and ATR) affects the therapeutic efficacy of a combination regimen consisting of PARPi and chemotherapeutics. Olaparib synergized with IT and OXA in a BRCA2-dependent manner (Figure 6A). Veliparib also increased the sensitivity to IT and OXA in a BRCA2-deficient background, which was generally not as strong as for olaparib. For the novel compounds, an additional cytotoxic effect was observed for the combination treatment with IT in HCT116 BRCA2^−/−^ cells but not with the other anticancer drugs (Figure 6B and Figure A6B). Consistently, bright field microscopy revealed morphological changes such as cell rounding and detachment in BRCA2-deficient HCT116 cells treated with X17613 and IT, indicative of cell death (Figure 6C).

**Figure A7:**
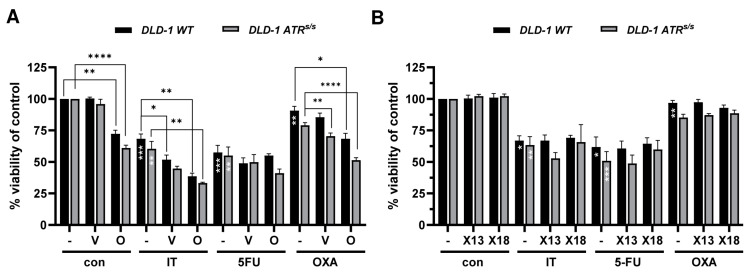
(**A**) Viability of DLD-1 WT and DLD-1 ATR^s/s^ cells after treatment with PARPi olaparib or veliparib in combination with chemotherapeutic drugs (IT, 2.5 µM), 5-fluorouracil (5-FU, 0.1 µM) and oxaliplatin (OXA, 5 µM) for 72 h (*n* ≥ 3). (**B**) Viability of DLD-1 WT and DLD-1 ATR^s/s^ cells after treatment with PARPi X17613 and X17618 in combination with chemotherapeutic drugs (IT, 2.5 µM), 5-fluorouracil (5-FU, 0.1 µM) and oxaliplatin (OXA, 5 µM) for 72 h (*n* ≥ 3). Data are shown as mean +/− SEM. * *p* < 0.01, ** *p* < 0.01, *** *p* < 0.001, **** *p* < 0.0001; *t*-test.

In order to investigate whether this effect is attributable to an increased DSB formation, we performed Western blot analysis of phosphorylated H2AX (γH2AX) as a well-established DSB marker [46]. To this end, HCT116 WT and BRCA2-deficient cells were pretreated with X17613 for 2 h and subsequently co-treated with IT for an additional 24 h. No significant increase in γH2AX was observed in HCT116 WT cells upon treatment with X17613 alone (Figure 6D,E). The genotoxic drug IT led to a significant induction of γH2AX as compared to the control. However, no significant further increase was detected after co-treatment as compared to IT mono-treatment. It should be noted that IT caused slightly higher γH2AX levels in BRCA2^−/−^ cells, which were further augmented in the presence of X17613 (Figure 6D,E), and that similar results were obtained for X17618 (Appendix A, Figure A6C). Further experiments in DLD-1 WT and DLD-1 ATR^s/s^ cells revealed little impact of the ATR status on the sensitivity of CRC cells towards a combination of PARPi and chemotherapeutic drugs (Appendix A, Figure A7A,B). Taken together, these findings provided evidence that PARPi (olaparib > veliparib > X17613 > X17618) and chemotherapeutic drugs (IT > OXA) synergize in a BRCA2-dependent manner in CRC cells.

## 4. Discussion

Our work addresses the role of PARP-1 as a target for chemotherapeutic intervention in both MSI and MSS CRC by applying novel PARP inhibitors to overcome PARPi resistance and reduce cytotoxicity-mediated adverse effects. Therefore, we analyzed an array of indole-based compounds by in silico screening regarding their ability to inhibit PARP-1 activity in a molecular docking model and used the compounds for in vitro testing by applying a cell-free assay based on recombinant PARP-1. The four substances with the lowest IC_50_ value and, thus, highest PARPi activity in vitro, X17613, X17618, X17620, and X17621, were also identified in silico to have the highest binding affinity to the active center of PARP-1. Interestingly, the compound, X17613, with the highest inhibitory activity contains a cyclic carboxylic acid hydrazide motif (i.e., a dihydrodiazepinone, [cd]-fused to indole). Among PARPi developed so far, a similar core structure can only be found in pamiparib, which is currently tested clinically for the treatment of brain tumors, since improved penetration across the blood–brain barrier was demonstrated [47]. Compared to X17613, olaparib and veliparib share key interactions with the amino acid residues G863, S904, and Y907 but contain an additional side chain that interacts with Y896 and D766, presumably leading to a higher potency. Further chemical modification of the lead compound X17613 might, thus, result in higher inhibitory activity. In a recent study, the side chain modification of veliparib was shown to drastically increase its PARP trapping activity due to allosteric retention, independent of enzymatic inhibition [48]. However, one should keep in mind that this will very likely also cause higher toxicity in healthy tissue, since the capability for PARP trapping closely correlates with PARPi toxicity [49].

Applying a CRC cell-based assay, the inhibitory activity of the four compounds was assessed by PAR staining and confocal microscopy. We detected a marked inhibition of H_2_O_2_-dependent PAR generation by X17613 and X17618, albeit at higher concentrations as compared to the positive control olaparib. The reduced activity of X17620 in the cell-based assay, despite potent inhibition of recombinant PARP-1, could be attributable to an efficient cellular efflux. Certain PARPi (e.g., olaparib) are known substrates for P-glycoprotein, which is an efflux transporter responsible for the resistance of cancers to numerous drugs, whereas other PARPi (e.g., veliparib) are not excreted [50].

We then assessed the ability of the novel PARPi to cause PARP-1 trapping in HCT116 and Caco-2 cells, which represent CRC cell models with MSI and MSS, respectively [51]. While olaparib induced substantial PARP-1 trapping, X17613 and X17618 showed no effects in both CRC cell models. Zandarashvili and colleagues dissected the molecular mechanisms that determine PARPi-dependent trapping of PARP-1 [48]. Catalytic inhibition of PARP-1 prevents automodification-dependent release, and, therefore, depends on the IC_50_ of the PARPi. Simultaneously, PARPi can influence PARP-1 allostery, which either promotes the release or retention of the enzyme, depending on its structure [48]. Veliparib was shown to have an allosteric pro-release effect, which olaparib is lacking [48]. It is conceivable that X17613 and X17618 also display an allosteric pro-release effect, which together with their lower PARPi activity compared to olaparib might explain the observed lack of PARP-1 trapping.

Interestingly, monotreatment with the compounds X17613 and X17618 as well as veliparib failed to induce cytotoxicity in HCT116 PARP-1^+/+^ and PARP-1^−/−^ cells. These three compounds were also not cytotoxic in nonmalignant HCEC. Only olaparib led to a decrease in viability in HCT116 PARP-1^+/+^ cells, and to a lesser degree also in HCT116 PARP-1^−/−^ cells, indicating the importance of PARP-1 trapping for cytotoxicity in cells without genetic susceptibility. It should be mentioned that mutations of PARP-1, which impair its DNA binding affinity and, thus, reduce cytotoxic PARP trapping, were observed in tumors with acquired PARPi-resistance, underlining the role of PARP trapping in a clinical setting [52]. As mentioned above, olaparib also decreased the viability in PARP-1 deficient HCT116 cells. This is likely attributable to the effects of olaparib on other PARPs, particularly PARP-2, which is inhibited with similar potency as PARP-1 (IC_50_ 56 nM vs. 13 nM) [53] and is trapped on DNA by olaparib via an allosteric pro-retention effect [54]. Furthermore, this could be explained by potential off-target effects of PARPi. Rucaparib and niraparib were, for example, shown to modulate cellular kinases in vitro at higher nanomolar concentrations [53,55].

In HCT116 BRCA2^−/−^ cells, which were used as a model for synthetic lethality by PARPi, the low cytotoxicity of X17613 and X17618, despite PARP-1 inhibition, might be caused by an insufficient inhibitor potency. While olaparib and veliparib restrained recombinant PARP-1 activity in low nM concentrations, a significant reduction in HCT116 BRCA2^−/−^ cell viability by these two PARPi could only be observed at around 100-fold higher concentrations. Since inhibition of recombinant PARP-1 by X17613 and X17618 occurs with 10–100-fold less potency as compared to olaparib and veliparib, equally higher doses should be necessary to induce cytotoxicity and might, therefore, not have been detected in our assays.

Nevertheless, the inhibitor X17613 led to a significant reduction in viability in HCT116 BRCA2^−/−^ cells in combination with the established cytostatic drug IT. These results show that the occurrence of HR deficiency renders CRC cells susceptible towards dual inhibition of TOP-1 and PARP-1, also in the absence of PARP-1 trapping. BRCA2 mutations in CRC are rare, occurring with a prevalence of around 1% [56], but were shown to be associated with an early onset of the disease [9]. Inhibition of PARP-1 could improve CRC chemotherapy beyond defects of BRCA1/2, leading to synergistic cytotoxicity in tumor cells. This was illustrated by siRNA-mediated knockdown of the HR-mediator RAD51 in colon cancer cells, which potentiated the cytotoxicity of olaparib monotreatment and in combination with SN38 [57]. Our study revealed a marked decrease in CRC cell viability due to co-treatment with olaparib or veliparib and the DNA-damaging agent IT. These results are in accordance with an earlier study conducted in human prostate cancer and glioblastoma cells, revealing synergistic effects of both PARPi with the TOP-1 inhibitor camptothecin [58]. The synergism between PARPi and TOP-1 inhibitors was further demonstrated using a HCT116 xenograft model, in which a combination regimen of IT and rucaparib strongly reduced tumor growth in vivo [59]. Furthermore, combinations of PARPi with inhibitors of ATR could provide a therapeutic approach for the treatment of tumors with or without HR deficiencies in the future, as shown in ovarian cancer models [60].

Interestingly, a comparison of HCT116 PARP-1^−/−^ and PARP-1^+/+^ revealed a higher cytotoxicity of the agents IT and OXA in the absence of PARP-1, highlighting its pivotal role in DNA repair and replication stress response. Consistent with this finding, we could detect a synergistic activity of olaparib with IT and OXA, but not with 5-FU in HCT116 PARP-1^+/+^ cells. These effects were generally also observed in DLD-1 cells, whereas in Caco-2 cells PARPi had little impact on the cytotoxic activity of the anticancer drugs. Applying different cancer cell lines, Murai et al. observed that enzymatic PARP inhibition is sufficient for synergistic effects by combination therapy with TOP-1 Inhibitors (see above), while PARP trapping is necessary for alkylating agents such as temozolomide [58]. We were able to confirm these results for CRC cells, showing that veliparib, which lacks PARP trapping activity, fails to induce cytotoxicity in combination with OXA, in contrast to olaparib. While inhibitor-induced PARP trapping is the main contributor to cytotoxicity in a monotherapy regimen, these observations imply that enzymatic inhibition is sufficient for the synergistic effect in combination with IT. This could allow for the application of better-tolerated PARPi in a combination treatment regimen. Hopkins et al. revealed PARP-1 trapping to be the primary mechanism of PARPi cytotoxicity towards healthy bone marrow cells [49]. This side effect was also found to limit the efficacy of olaparib for CRC treatment in a clinical study by further amplification of the adverse effects of chemotherapeutic drugs in a combination regimen [23]. It might, therefore, be worth considering the application of PARPi with attenuated PARP trapping activity in combination with TOP-1 inhibitors to reduce dose-limiting toxicity (DLT). This is an important clinical aspect since TOP-1 inhibitors, like IT, also cause myelosuppression as DLT. Genetic variants of the phase II gene *UDP-glucuronosyltransferase 1A1 (UGT1A1)* were clearly linked to severe myelosuppression and neutropenia [61]. Diarrhea represents the other most common DLT in response to IT administration. This can occur rapidly within the first hours or in a delayed manner after 24 h, representing a very serious and potentially life-threatening situation [62]. Indeed, the combination of PARPi with the TOP-1 inhibitors IT and topotecan for the treatment of patients with solid tumors was illustrated as challenging due to the severe myelosuppression and diarrhea. However, our new PARPi compounds with little cytotoxicity due to the lack of PARP trapping might be a promising alternative for the combination therapy with TOP-1 inhibitors. Furthermore, it was proposed to switch the combination therapy regimen to a gapped schedule, which avoids the overlapping toxicity of PARPi and TOP-1 inhibitors in normal healthy tissue such as bone marrow [62]. Another possibility in this regard might be the use of indenoisoquinolines, which are TOP-1 inhibitors structurally unrelated to camptothecin and irinotecan [63]. DLD-1 cells with *BRCA2* deficiency were hypersensitive towards these compounds, which further synergized with olaparib in vitro and in vivo [63].

Moreover, PARPi and other DDR inhibitors such as ATRi have also great potential as radiosensitizers, which is of particular interest for the treatment of rectal cancer [64]. Radiotherapy represents an important treatment modality for rectal cancer, typically performed in combination with chemotherapy in a peri- or postoperative setting depending on the tumor and nodes stages [65]. Using cell culture models and an in vivo xenograft mouse model, olaparib was shown to sensitize CRC cells (i.e., HCT116 and SW480) towards radiotherapy in a XRCC2-dependent manner [66]. Furthermore, the PARPi talazoparib synergized with radiotherapy in CRC cells with both wild-type BRAF (i.e., DLD-1) and mutant BRAF (i.e., RKO), while olaparib had lower synergistic effects [67]. In addition to those preclinical studies, a phase 1b clinical study was conducted in patients with locally advanced rectal cancer. This investigated the safety and tolerability of veliparib in combination with chemoradiotherapy (CRT), consisting of the orally available 5-FU prodrug capecitabine and fractionated radiotherapy [68]. Interestingly, the results provided first evidence that veliparib could potentiate the antitumor activity of CRT [68]. It is, therefore, tempting to speculate that our novel identified PARPi X17613 and X17618 may also sensitize CRC cells towards radiotherapy or radiotherapy combined with IT, which warrants future preclinical studies. There are completed clinical studies showing a lack of PARPi efficacy, in which CRC patients were not stratified according to their PARP-1 expression beforehand [23,69]. Routine assessment of PARP-1 expression in CRC biopsies is, therefore, strongly recommended to identify patients who might benefit from PARPi combination therapy, while patients with low PARP-1 expression might be suitable for application of other DDR inhibitors, with ATR [70] and RAD51 [71] as promising targets. More recently, a composite biomarker approach has been described to predict responses to ATR inhibitors. This is based upon the detection of basal pSer33-RPA32 levels, RAD51 foci, ATM, and RAD51C expression in formalin-fixed paraffin-embedded colorectal tumor samples or derived preclinical models [72]. It is obvious to complement this set of biomarkers by PARP-1 expression in order to select the tailored cancer therapy regimen.

## 5. Conclusions

In summary, our study identified novel PARPi lead structures with potent PARP-1 inhibitory activity in CRC cells but low cytotoxicity in wildtype CRC cells and no adverse effects on normal HCEC due to lack of PARP trapping. The most promising compound, X17613, synergized with the anticancer drug and TOP-1 inhibitor IT in a BRCA2-dependent manner, which can be transferred to other settings with HRD. In support of this view, a very recent study provided evidence that PARP inhibition, rather than PARP trapping, is sufficient for killing cancer cells with HRD [73].

## Figures and Tables

**Table 1:** IC_50_-values [µM] of established PARP inhibitors (olaparib and veliparib), two PARP inhibitor candidates (X17613 and X17618) and chemotherapeutic drugs in CRC cells with or without DDR defects. “-“ indicates that IC_50_ values could not be determined due to insufficient or lack of cytotoxicity.

	X17613	X17618	Olaparib	Veliparib	IT	5-FU	OXA
HCT116 WT	-	-	8.40	-	1.28	0.50	1.19
HCT116 BRCA2^−/−^	-	-	0.46	7.45	0.16	0.34	0.25
HCT116 PARP1^+/+^	-	-	11.21	-	0.93	0.24	0.71
HCT116 PARP1^−/−^	-	-	-	-	0.35	0.15	0.28
DLD-1 WT	-	-	5.88	-	4.19	0.35	8.77
DLD-1 ATR^s/s^	-	-	1.99	-	2.99	0.22	9.08
Caco-2	-	-	-	-	66.52	-	11.18
HCEC	-	-	-	-	2.95	26.23	40.71

## Data Availability

The data generated during this study were included in the article and its supplementary files. All data on the synthesis and analysis of the main compounds X17613, X17618, X17620, and X17621 are also available via the Chemotion repository (https://www.chemotion-repository.net/) and can be accessed via the following DOI: https://dx.doi.org/10.14272/collection/CWG_2024-02-14 (accessed on 14 February 2024) [74].

## Supplementary Materials

The following supporting information can be downloaded at: https://www.mdpi.com/article/10.3390/cancers16203441/s1, Supplementary Material and Methods: synthesis of test compounds. Scheme S1: Synthesis of compound 4; Scheme S2: Synthesis of compounds 10a–c. Figure S1: ^1^H-NMR of 1-oxo-2,6-dihydro-1H-[1,2]diazepino [4,5,6-cd]indole-4-carboxylic acid (X17613); Figure S2: ^13^C-NMR of 1-oxo-2,6-dihydro-1H-[1,2]diazepino [4,5,6-cd]indole-4-carboxylic acid (X17613); Figure S3: ^1^H-NMR of N,2-dimethyl-5-oxo-1,3,4,5-tetrahydrobenzo[cd]indole-3-carboxamide (X17618); Figure S4: ^13^C-NMR of N,2-dimethyl-5-oxo-1,3,4,5-tetrahydrobenzo[cd]indole-3-carboxamide (X17618); Figure S5: ^1^H-NMR of N-(furan-2-ylmethyl)-2-methyl-5-oxo-1,3,4,5-tetrahydrobenzo[cd]indole-3-carboxamide (X17620); Figure S6: ^13^C-NMR of N-(furan-2-ylmethyl)-2-methyl-5-oxo-1,3,4,5-tetrahydrobenzo[cd]indole-3-carboxamide (X17620); Figure S7: ^1^H-NMR of 2-methyl-3-(morpholine-4-carbonyl)-3,4-dihydrobenzo[cd]indol-5(1H)-one (X17621); Figure S8: ^13^C-NMR of 2-methyl-3-(morpholine-4-carbonyl)-3,4-dihydrobenzo[cd]indol-5(1H)-one (10c) (X17621); Figure S9: Uncropped Western blot images of HCT116 cells shown in Figure 3A (#1) and two independent repetitions (#2 and #3); Figure S10: Uncropped Western blot images of Caco-2 cells shown in Figure 3B (#1) and two independent repetitions (#2 and #3); Figure S11: Uncropped Western blot images of HCT116 WT and BRCA2^−/−^ cells shown in Figure 6C (#1) and three independent repetitions (#2–#4); Figure S12: Uncropped Western blot images of HCT116 WT, HCT116 BRCA2^−/−^, HCT116-PARP1^+/+^, HCT116 PARP-1^−/−^, DLD-1 WT and DLD-1 ATR^s/s^ cells shown in Figure A5; Figure S13: Uncropped Western blot images of HCT116 WT and BRCA2^−/−^ cells shown in Figure A6C (#1) and three independent repetitions (#2–#4).
