# Supplementary material for: Targeting PARP-1 and DNA Damage Response Defects in Colorectal Cancer Chemotherapy with Established and Novel PARP Inhibitors
Source: Cancers (Basel). 2024 Oct 10;16(20):3441. doi: 10.3390/cancers16203441 (PMC11506018; doi:10.3390/cancers16203441)
Supplement: Supplementary file 1 [file cancers-16-03441-s001.zip › cancers-3204604-supplementary.pdf]

cancers MDPI

SUPPORTING INFORMATION – MATERIAL AND METHODS

Synthesis of test compounds

Synthesis of test compound **4** (X17613, s. scheme 1) was conducted as described below. The reaction sequence started with the acylation of the starting material methyl 1*H*-indole-4-carboxylate (**1**) by treatment with oxalyl chloride in the cold to achieve acylation of the indole heterocycle in the 3-position. The resulting 3-(2-chloro-2-oxo-acetyl)oxy-indole derivative **2** was then subjected to hydrolysis of the acyl chloride motif by addition of water (in THF) to yield the free 2-oxoacetic acid **3**at ambient temperature. Due to the almost quantitative character of most reactions in this cascade and the high reactivity of key intermediates, isolation and analytical characterization of these intermediates was omitted, as reported for similar structural motifs [1,2]. During the final step, cyclization of **3** to **4** was achieved by means of reacting the substrate **3** with aqueous hydrazine hydrate solution in the presence of acetic acid. Following this procedure, compound **4** (X17613) could be isolated as a yellow amorphous solid with a yield of 76% over three steps (S. Scheme S1).

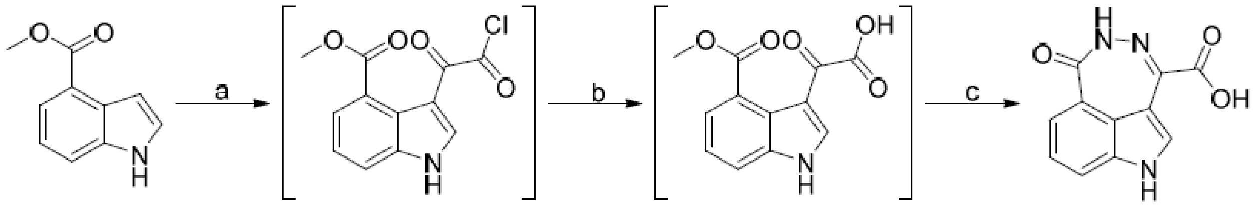

Scheme S1. Synthesis of compound **4**. Reagents and conditions: (a) oxalyl chloride, DEE, 0–20 °C, 12 h; (b) H_2O, THF, 20 °C, 2 h; (c) N_2H_4 x H_2O, CH_3COOH, MeOH, 20 °C, 18 h, 76% (over three steps).

Compounds **10a-c** (X17618, X17620, X17621) were synthesized from their respective carboxylic acid and amine precursors via HATU/DIPEA-assisted amide coupling reactions. The corresponding 1,3,4,5-tetrahydrobenzo[*cd*]indole-derived carboxylic acid **9** was prepared by adapting a procedure from Böshagen et al. (s. Scheme S2) [3]. Thus, the starting material 2-methylindole (**5**) was reacted withmaleic acid to yield the intermediate succinic acid derivative **6**, which was dehydrated in the next step using prop-1-en-2-yl acetate and *p*-toluenesulfonic acid as the catalyst. Due to the ability of prop-1-en-2-yl acetate to act as an acetylating agent, the N-atom of the indole nucleus was acetylated as a side reaction, which did, however, not pose any problems regarding the following steps. Formation of the tricyclic carboxylic acid **8** was achieved via intramolecular Friedel–Crafts acylation using dry aluminum chloride in 1,2-dichloroethane with excellent yields. Removal of the accidentally introduced *N*-acetyl group of **8** proceeded smoothly by alkaline hydrolysis, followed by precipitation of the carboxylic acid **9**. With the key intermediate **9** thus prepared, derivatization to amides like **10** by reacting it with various amines proved to be fairly straightforward. Standard amidecoupling reagents HATU and DIPEA in DMF were utilized to achieve this goal. The reaction times could be shortened significantly by reacting the mixture in a microwave reactor at elevated temperatures (18 h at 20°C vs. 10 min at 140°C and microwave irradiation).

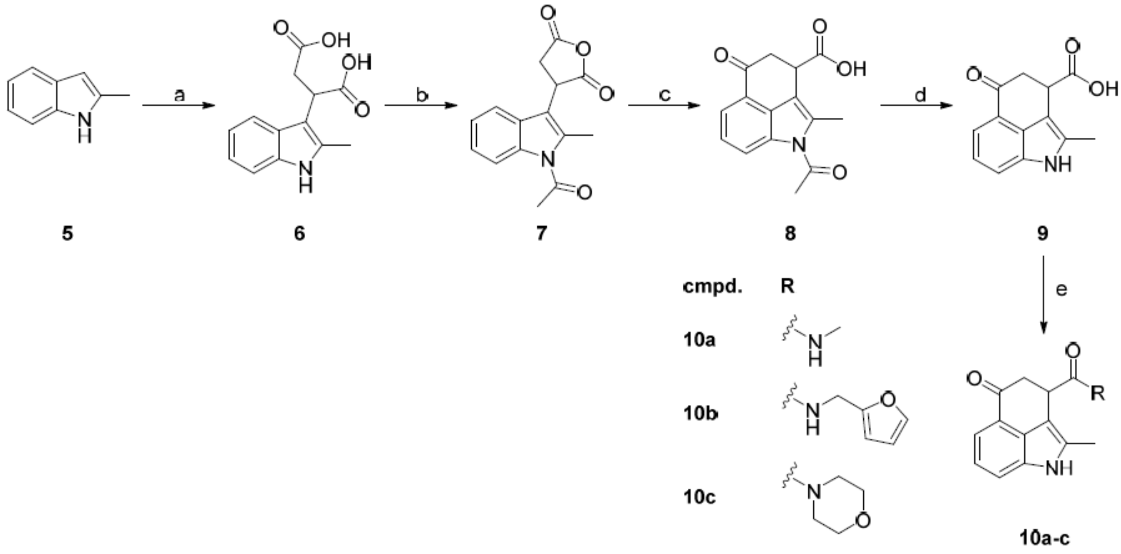

Scheme S2. Synthesis of compounds **10a-c**. Reagents and conditions: (a) maleic acid, 120 °C, 1 h, 83%; (b) prop-1-en-2-yl acetate, TsOH, 20-120 °C, 61%; (c) $AlCl_3$, DCE, 50 °C, 94%; (d) NaOH, H_2O, 75 °C, 87%; (e) R_1NHR_2, HATU, DIPEA, DMF, 140 °C, 10 min, µ-wave irradiation, 35-56%.

Experimental Section

General Remarks

All starting materials, reagents, and solvents were commercially available and purchased from Sigma-Aldrich, VWR, abcr, or Carl Roth. Unless otherwise stated, starting materials were used as provided. Melting points were determined using a BÜCHI Melting Point M-565 device. Analytical thin-layer chromatography was performed using silica gel 60 F254 aluminum plates supplied by Merck; visualization was accomplished with UV light. Microwave-assisted syntheses were carried out in a Monowave 400 reactor manufactured by Anton Paar in suitable glass vessels while monitoring and controlling the temperature with the integrated infrared sensor. Preparative column chromatography was carried out with an Interchim puriFlash XS 520Plus system and the corresponding 25 g silica gel cartridges available from Interchim (30-SI-HP). NMR analysis was run on a Bruker Avance III instrument at 400 MHz (^{1}H) and 101 MHz (^{13}C), using DMSO-*d6* as the solvent. Chemical shifts are given in relation to the internal standard tetramethylsilane and reported as parts per million (ppm). MIR analyses were performed on an ALPHA-FT-IR device from Bruker Optics equipped with a diamond ATR accessory unit. High-resolution (HR) accurate mass (AM)-MS analyses were performed either on a Bruker MAXIS LC-QTOF-MS or a Bruker compact LC-qQTOF-MS run with ESI ionization.

Synthesis

1. -Oxo-2,6-dihydro-1H-[1,2]diazepino[4,5,6-cd]indole-4-carboxylic acid (4) (X17613)

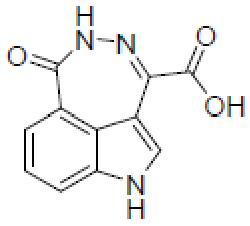

Synthetic procedure modified from Ferraris et al. and Webber et al. [1,2]. To a round-bottom flask were added methyl 1*H*-indole-4-carboxylate (**1**, 3.50 g, 20.00 mmol, 1.00 eq) and 100 mL diethyl ether. To the suspension, oxalyl chloride (3.05 g, 2.06 mL, 24.00 mmol, 1.20 eq) was added dropwise via an addition funnel. After stirring at 20 °C for 12 h, the solvent was evaporated, and the residue resuspended in 75 mL of tetrahydrofuran, followed by the addition of water (1.80 mL, 1.80 g, 100.00 mmol, 5.00 eq). After stirring at 20 °C for 2 h, the solvent was evaporated. The residue was resuspended in methanol (12 mL) and glacial acetic acid (1.72 mL, 1.80 g, 30.00 mmol, 1.50 eq) and an aqueous solution of hydrazine hydrate (3.8 mL, equals 3.00 g of hydrazine hydrate, 60.00 mmol, 2.00 eq) were added. The mixture was stirred at 20 °C for 18 h. The precipitate was separated via vacuum filtration, washed multiple times with water, and dried under reduced pressure, yielding **4** as a yellow amorphous solid (3.50 g, 76%).

R_f = 0.62 (15% acetic acid and 15% water in *n*-butanol); mp: 226.8°C; ^{1}H-NMR (400 MHz, DMSO-d_6): δ (ppm) = 7.05-7.16 (m, 1H), 7.41-7.61 (m, 2H), 7.89 (s, 1H), 10.00 (s, 1H), 11.89 (s, 1H); ^{13}C-NMR (101 MHz, DMSO-d6): δ (ppm) = 110.9, 116.1, 120.5, 121.9, 123.4, 127.2, 128.1, 128.6, 135.9, 165.2, 167.9; IR (ATR): $\tilde{\nu}$ = 3222 (m, $\nu_{N\text{-}H}$), 3098 (m, $\nu_{O\text{-}H}$), 1669 (s, $\nu_{C=O}$); ESI-HRAM-MS (*m*/*z*): calcd.for $[C_{11}H_7N_3O_3 + H]^+$ 230.0560, found 230.0559.

Additional information on the chemical synthesis is available via the Chemotion repository: https://dx.doi.org/10.14272/reaction/SA-FUHFF-UHFFFADPSC-DRRJDSUMVP-UHFFFADPSC-NUHFF-NUHFF-NUHFF-ZZZ

Additional information on the analysis of the target compound is available via the Chemotion repository: https://dx.doi.org/10.14272/DRRJDSUMVPRFOC-UHFFFAOYSA-N.2

N,2-Dimethyl-5-oxo-1,3,4,5-tetrahydrobenzo[cd]indole-3-carboxamide (10a) (X17618)

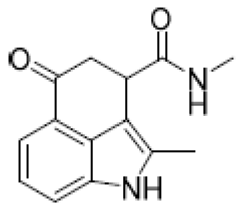

To a G10 microwave reaction vial were added **9** (114 mg, 0.50 mmol, 1.00 eq), methylamine hydrochloride (34 mg, 0.50 mmol, 1.00 eq), HATU (380 mg, 1.00 mmol, 2.00 eq), DIPEA (610 µL, 452 mg, 3.50 mmol, 7.00 eq), and dry DMF (4.0 mL). The vial was flushed with argon and the mixture was heated in a microwave reactor at 140 °C for 10 min. It was poured into water and extracted multiple times with ethyl acetate. The combined organic phases were washed with diluted hydrochloric acid (1M) and saturated aqueous sodium chloride solution. The product was isolated via column chromatography using an *n*-hexane/ethyl acetate gradient, yielding an amorphous yellow solid (35 mg, 35%).

R_f = 0.27 (50% ethyl acetate in *n*-hexane); mp: 248.3°C (decomp.); ^{1}H-NMR (400 MHz, DMSO-d_6): δ (ppm) = 2.39 (s, 3H), 2.56 (d, *J* = 4.6 Hz, 3H), 2.73 (dd, *J* = 16.0 Hz, *J* = 3.9 Hz, 1H), 2.85 (dd, *J* = 16.0 Hz, *J* = 6.6 Hz, 1H), 4.04 (dd, *J* = 6.6 Hz, *J* = 3.9 Hz, 1H), 7.07-7.12 (m, 1H), 7.28 (dd, *J* =7.4 Hz, *J* = 0.6 Hz, 1H), 7.45 (dd, *J* = 7.9 Hz, *J* = 0.6 Hz, 1H), 7.97 (d, *J* = 4.6 Hz, 1H), 11.07 (s, 1H); ^{13}C-NMR (101 MHz, DMSO-d6): δ (ppm) = 11.6, 25.6, 39.5, 42.3, 105.1, 113.7, 114.9, 120.6, 124.0, 132.8, 132.8, 134.0, 172.9, 196.0; IR (ATR): $\tilde{\nu}$ = 3317 (m, $\nu_{N\text{-}H}$), 2916 (w, $\nu_{C\text{-}H}$), 1640 (s, $\nu_{C=O}$); ESI- HRAM-MS (*m*/*z*): calcd. for $[C_{28}H_{28}N_4O_3 + H]^+$ ($[2M+H]^+$) 485.2183, found 485.2201.

Additional information on the chemical synthesis is available via the Chemotion repository:

https://dx.doi.org/10.14272/reaction/SA-FUHFF-UHFFFADPSC-QJYPGCPJNL-UHFFFADPSC-NUHFF-NUHFF-NUHFF-ZZZ

Additional information on the analysis of the target compound is available via the Chemotion repository: https://dx.doi.org/10.14272/QJYPGCPJNLNOJT-UHFFFAOYSA-N.1

***N*-(Furan-2-ylmethyl)-2-methyl-5-oxo-1,3,4,5-tetrahydrobenzo[*cd*]indole-3-carboxamide (10b)(X17620)**

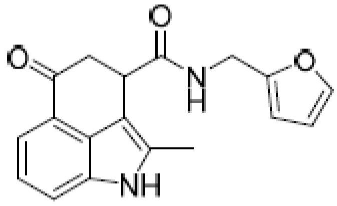

To a G10 microwave reaction vial were added **9** (172 mg, 0.75 mmol, 1.00 eq), furfurylamine (66 µL,73 mg, 0.75 mmol, 1.00 eq), HATU (570 mg, 1.50 mmol, 2.00 eq), DIPEA (653 µL, 485 mg, 3.75 mmol, 5.00 eq), and dry DMF (4.0 mL). The vial was flushed with argon and the mixture was heated in a microwave reactor at 140 °C for 10 min. It was poured into water and extracted multiple times with ethyl acetate. The combined organic phases were washed with diluted hydrochloric acid (1M) and saturated aqueous sodium chloride solution. The product was isolated via column chromatography using an *n*-hexane/ethyl acetate gradient, yielding an amorphous yellow solid (130 mg, 56%).

R_f = 0.53 (50% ethyl acetate in *n*-hexane); mp: 193.4 °C; ^{1}H-NMR (400 MHz, DMSO-d_6): δ (ppm) = 2.35 (s, 3H), 2.72 (dd, *J* = 16.0 Hz, *J* = 3.7 Hz, 1H), 2.88 (dd, *J* = 16.0 Hz, *J* = 6.7 Hz, 1H), 4.13 (dd,*J* = 6.7 Hz, *J* = 3.7 Hz, 1H), 4.20 (dd, *J* = 15.6 Hz, *J* = 5.8 Hz, 1H), 4.26 (dd, *J* = 15.6 Hz, *J* = 4.7 Hz,1H), 6.18 (dd, *J* = 3.2 Hz, *J* = 0.7 Hz, 1H), 6.38 (dd, *J* = 3.2 Hz, *J* = 1.9 Hz, 1H), 7.10 (t, *J* = 7.6 Hz, 1H), 7.28 (dd, *J* = 7.3 Hz, *J* = 0.5 Hz, 1H), 7.45 (dd, *J* = 7.3 Hz, *J* = 0.5 Hz, 1H), 7.57 (dd, *J* = 1.8 Hz, *J* = 0.8 Hz, 1H), 8.55 (t, *J* = 5.6 Hz, 1H), 11.06 (s, 1H); ^{13}C-NMR (101 MHz, DMSO-d6): δ (ppm)=11.5, 35.5, 39.4, 42.1, 104.9, 106.7, 110.4, 113.7, 114.9, 120.6, 123.9, 132.8, 132.9, 134.0, 142.1, 152.1, 172.4, 195.9; IR (ATR): $\tilde{\nu}$= 3307 (m, $\nu_{N\text{-}H}$), 1630 (s, $\nu_{C=O}$); ESI-HRAM-MS (*m*/*z*): calcd. for $[C_{18}H_{16}N_2O_3 + H]^+$ 331.1053, found 331.1053.

Additional information on the chemical synthesis is available via the Chemotion repository:

https://dx.doi.org/10.14272/reaction/SA-FUHFF-UHFFFADPSC-QIROZZVNYL-UHFFFADPSC-NUHFF-NUHFF-NUHFF-ZZZ

Additional information on the analysis of the target compound is available via the Chemotion repository:https://dx.doi.org/10.14272/QIROZZVNYLUVGF-UHFFFAOYSA-N.1

2. -Methyl-3-(morpholine-4-carbonyl)-3,4-dihydrobenzo[cd]indol-5(1H)-one (10c) (X17621)

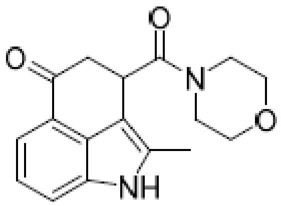

To a G10 microwave reaction vial were added **9** (172 mg, 0.75 mmol, 1.00 eq), morpholine (65 µL, 0.75 mmol, 1.00 eq), HATU (570 mg, 1.00 mmol, 2.00 eq), DIPEA (653 µL, 485 mg, 3.75 mmol, 5.00 eq), and dry DMF (4.0 mL). The vial was flushed with argon

and the mixture was heated in a microwave reactor at 140 °C for 10 min. It was poured into water and extracted multiple times with ethyl acetate. The combined organic phases were washed with diluted hydrochloric acid (1N) and saturated aqueous sodium chloride solution. The product was isolated via column chromatography using an *n*-hexane/ethyl acetate gradient, yielding an amorphous orange solid (83 mg, 37%).

R_f = 0.61 (50% ethyl acetate in *n*-hexane); mp: >300 °C; ^{1}H-NMR (400 MHz, DMSO-d_6): δ (ppm)=2.37 (s, 3H), 2.69 (dd, *J* = 15.8 Hz, *J* = 4.2 Hz, 1H), 2.83 (dd, *J* = 15.8 Hz, *J* = 6.2 Hz, 1H), 3.22-3.59 (m, 8H), 4.77 (dd, *J* = 5.9 Hz, *J* = 4.2 Hz, 1H), 7.10 (t, *J* = 7.6 Hz, 1H), 7.26-7.31 (m, 1H), 7.44 (d, *J* = 7.6 Hz, 1H), 11.06 (s, 1H); ^{13}C-NMR (101 MHz, DMSO-d6): δ (ppm) =12.2, 34.4, 42.7, 46.3, 66.3, 105.3, 113.7, 114.9, 120.6, 124.2, 132.2, 132.9, 134.0, 171.7, 195.9; IR (ATR): $\tilde{\nu}$= 3238 (m, $\nu_{N\text{-}H}$), 2856 (w, $\nu_{C\text{-}H}$), 1661 (s, $\nu_{C=O}$); ESI-HRAM-MS (*m*/*z*): calcd. for $[C_{17}H_{18}N_2O_3 + H]^+$ 321.1204, found 321.1204.

Additional information on the chemical synthesis is available via the Chemotion repository: https://dx.doi.org/10.14272/reaction/SA-FUHFF-UHFFFADPSC-AHZDMNVTLQ-UHFFFADPSC-NUHFF-NUHFF-NUHFF-ZZZ

Additional information on the analysis of the target compound is available via the Chemotion repository: https://dx.doi.org/10.14272/AHZDMNVTLQOTLN-UHFFFAOYSA-N.1

References

1. Ferraris, D.V., Li, J.H., Kalish, V.J., Zhang, J. Benzoazepine and benzodiazepine derivatives andtheir use as parp inhibitors. Patent WO0244183 (A2), 6 June 2002.
2. Webber, S.E., Canan, K.S.S., Tikhe, J., Thoresen, L.H. Tricyclic Inhibitors of Poly(ADP-ribose)polymerases. Patent WO0042040 (A1), 20 July 2000.
3. Böshagen, H., Rosentreter, U., Perzborn, E., Fiedler, V. Tetrahydro-1-benzo-(c,d)- indolpropionsäure-sulfonamide. Patent DE3826371 (A1), 8 February 1990.

SUPPORTING INFORMATION – FIGURES AND FIGURE LEGENDS

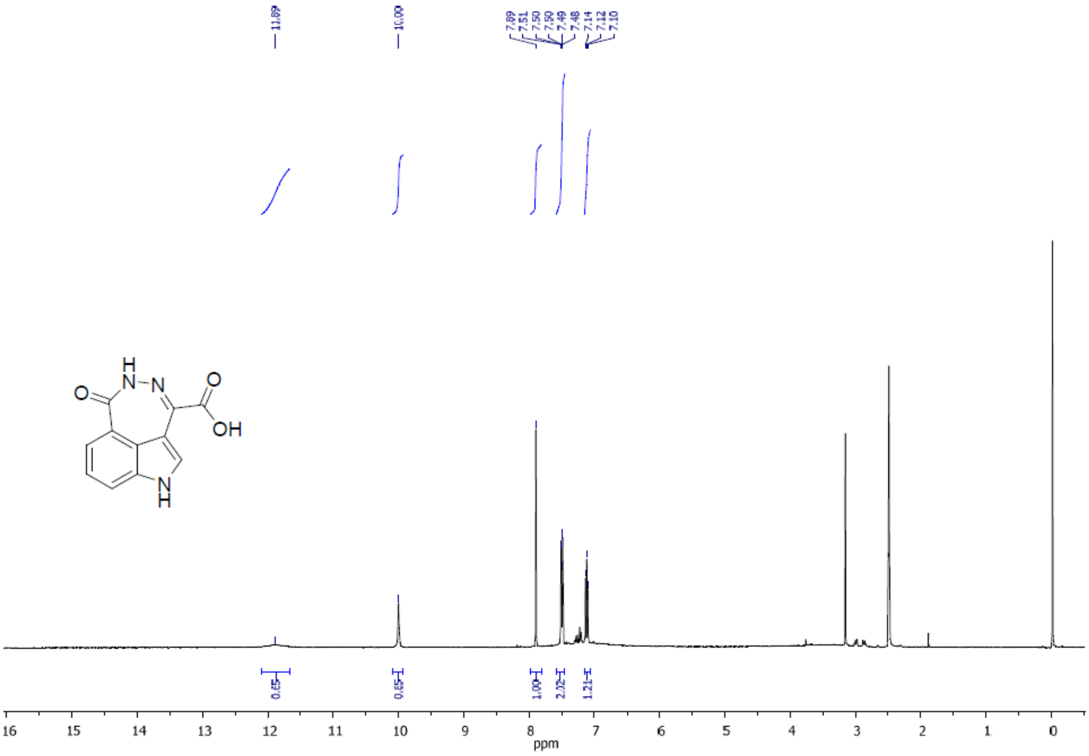

Figure S1. ^{1}H-NMR spectrum of 1-oxo-2,6-dihydro-1*H*-[1,2]diazepino[4,5,6-*cd*]indole-4-carboxylic acid (**4**) (X17613).

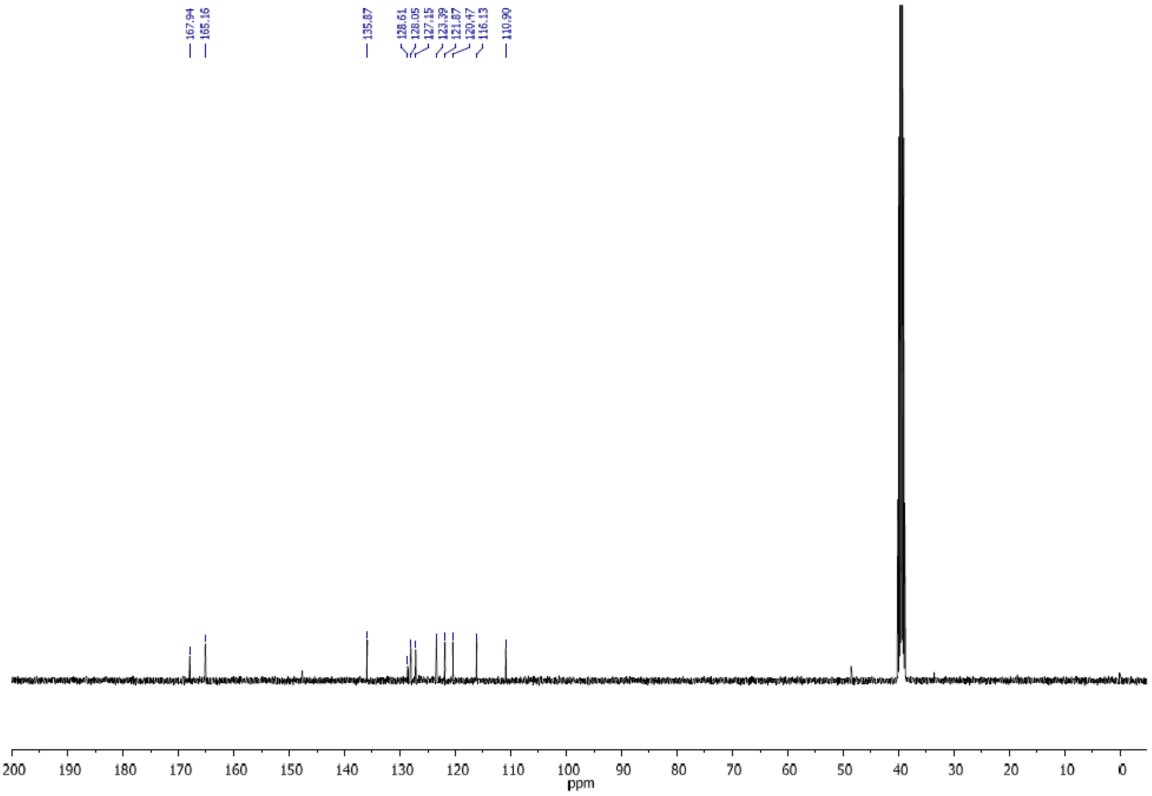

Figure S2. ^{13}C-NMR spectrum of compound 1-oxo-2,6-dihydro-1*H*-[1,2]diazepino[4,5,6-*cd*]indole-4-carboxylic acid (**4**) (X17613).

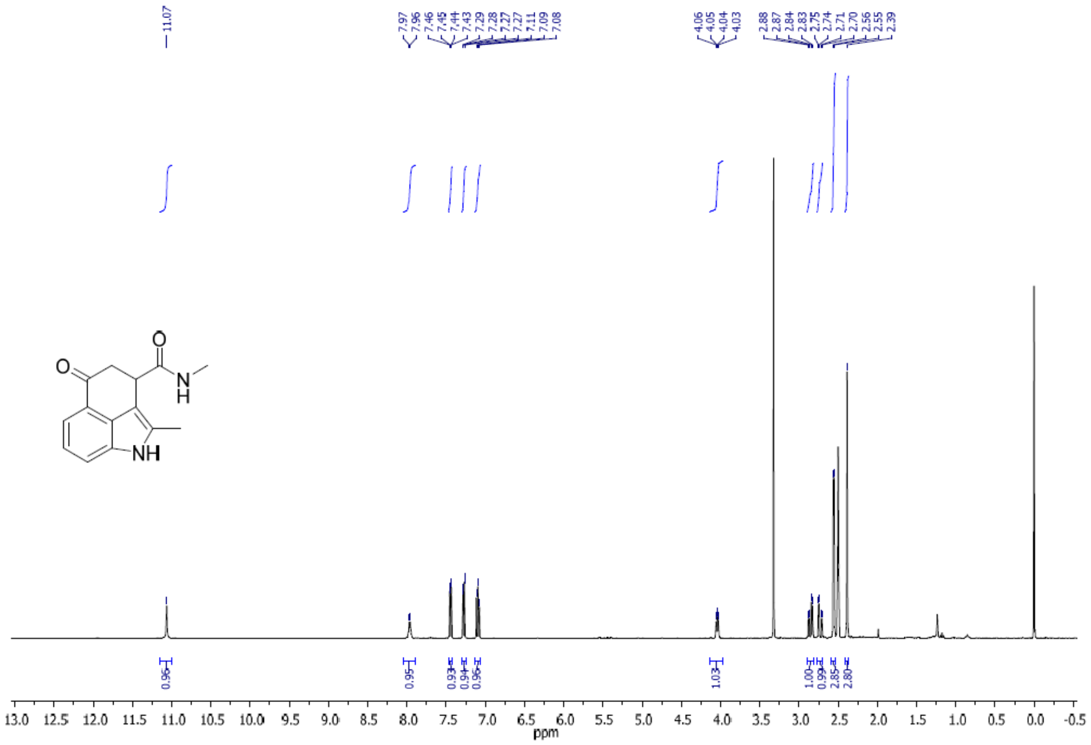

Figure S3. ^{1}H-NMR spectrum of *N*,2-dimethyl-5-oxo-1,3,4,5-tetrahydrobenzo[*cd*]indole-3-carboxamide (**10a**)(X17618).

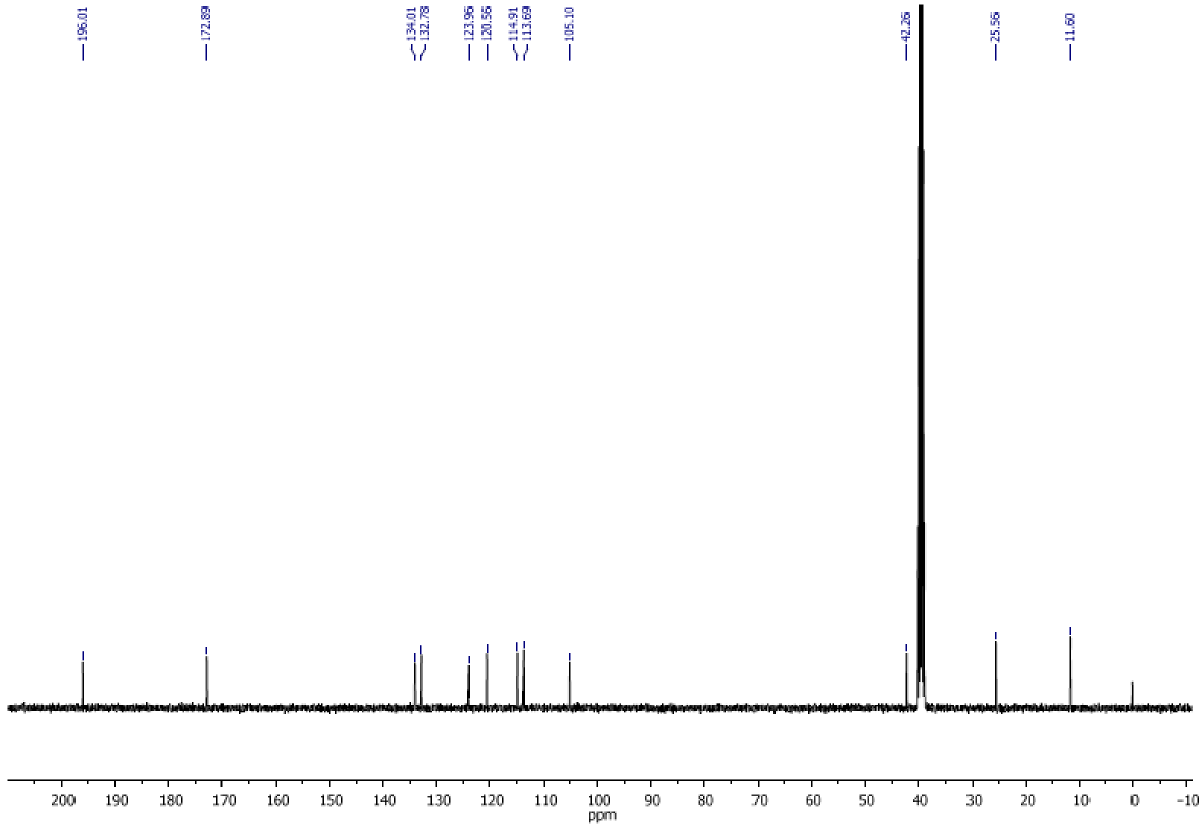

Figure S4. ^{13}C-NMR spectrum of *N*,2-dimethyl-5-oxo-1,3,4,5-tetrahydrobenzo[*cd*]indole-3-carboxamide (**10a**)(X17618).

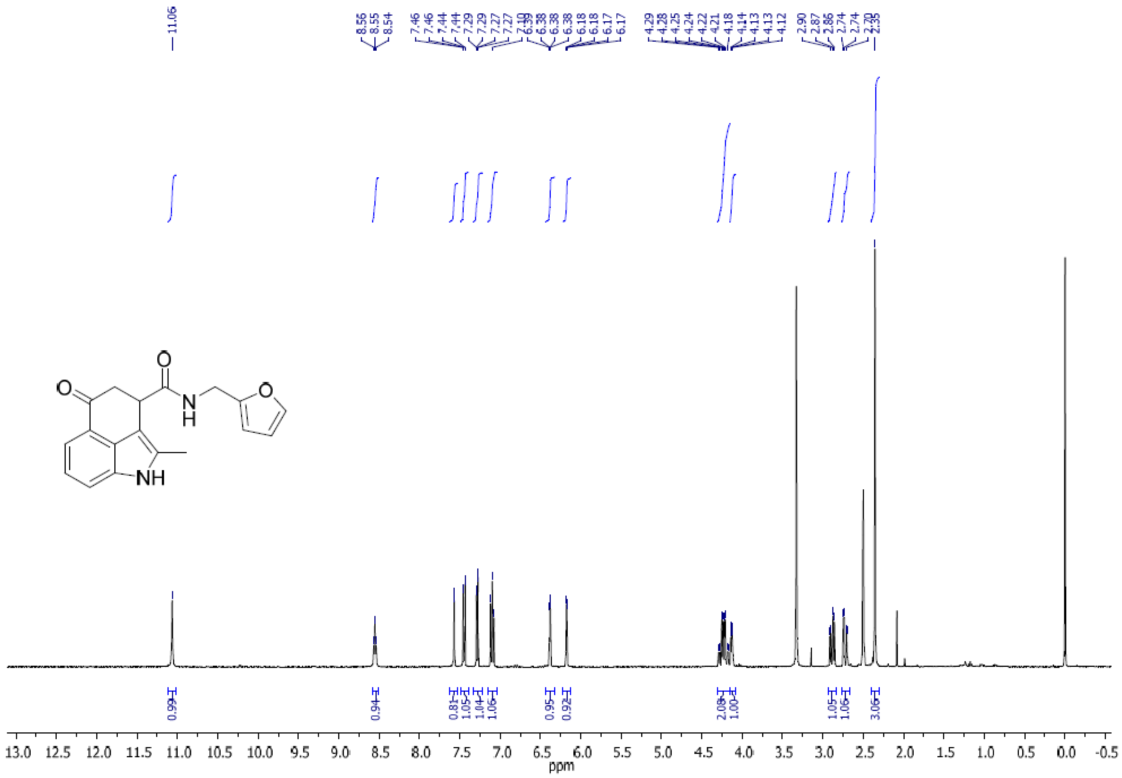

Figure S5. ^{1}H-NMR spectrum of *N*-(furan-2-ylmethyl)-2-methyl-5-oxo-1,3,4,5-tetrahydrobenzo[*cd*]indole-3- carboxamide (**10b**) (X17620).

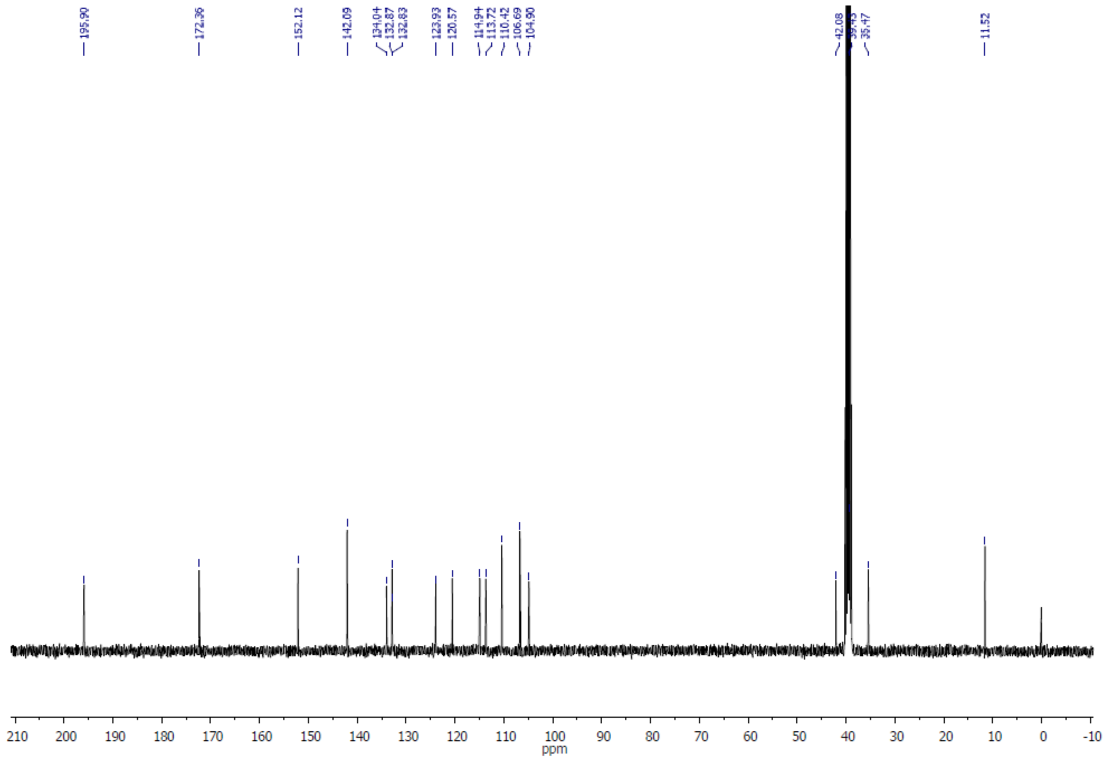

Figure S6. ^{13}C-NMR spectrum of *N*-(furan-2-ylmethyl)-2-methyl-5-oxo-1,3,4,5-tetrahydrobenzo[*cd*]indole-3- carboxamide (**10b**) (X17620).

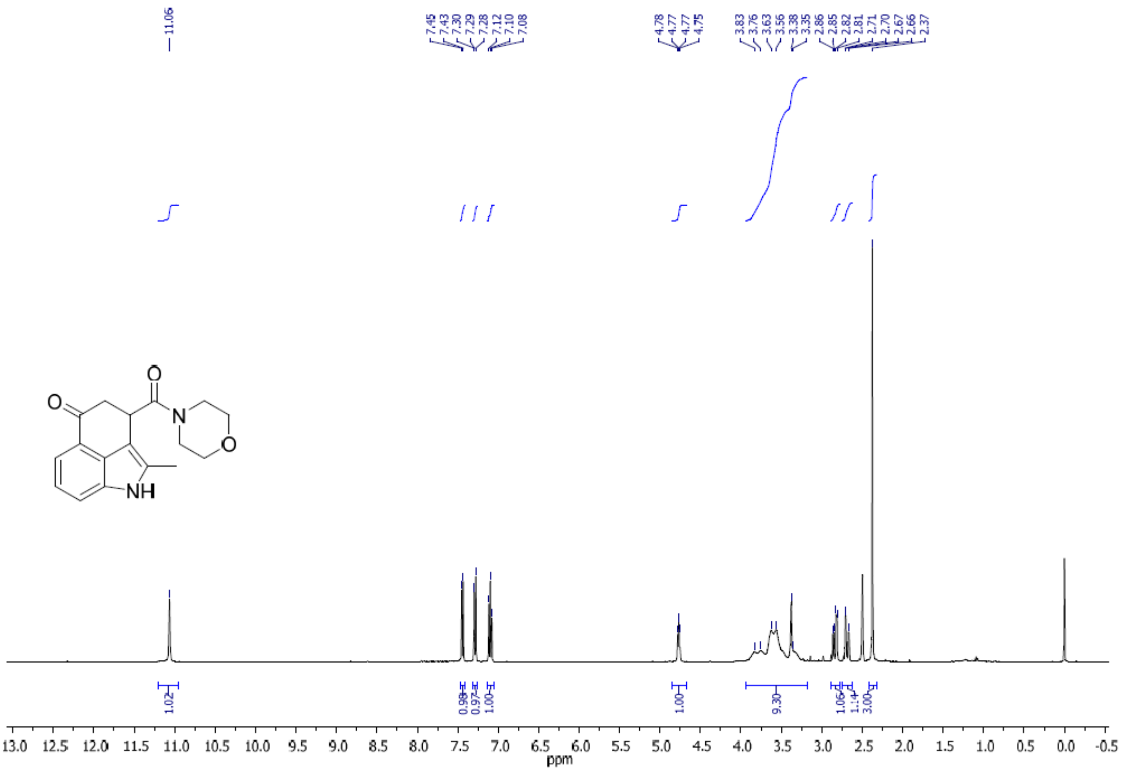

Figure S7. ^{1}H-NMR spectrum of 2-methyl-3-(morpholine-4-carbonyl)-3,4-dihydrobenzo[*cd*]indol-5(1*H*)-one(**10c**) (X17621).

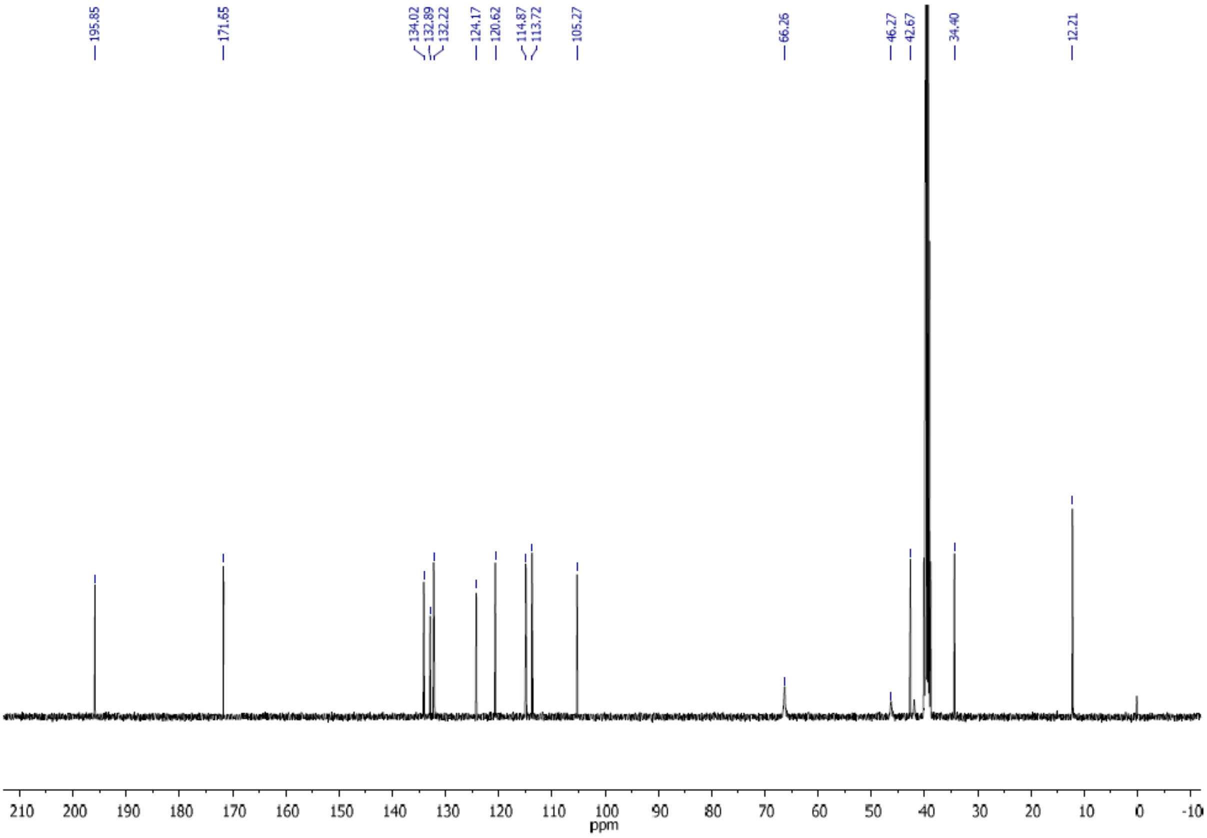

Figure S8. ^{13}C-NMR spectrum of 1H-NMR spectrum of 2-methyl-3-(morpholine-4-carbonyl)-3,4-dihydrobenzo[*cd*]indol-5(1*H*)-one (**10c**) (X17621).

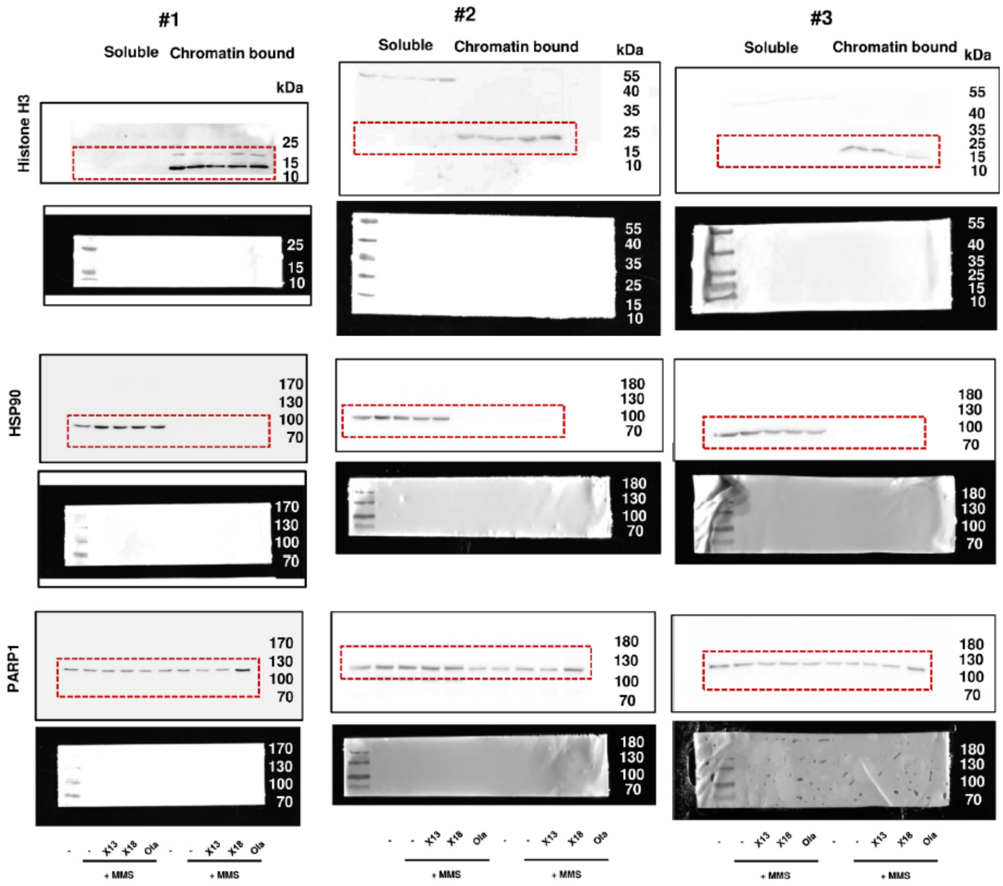

Figure S9. Uncropped Western blot images of HCT116 cells shown in Figure 3A (#1) and two independent repetitions (#2 and #3).

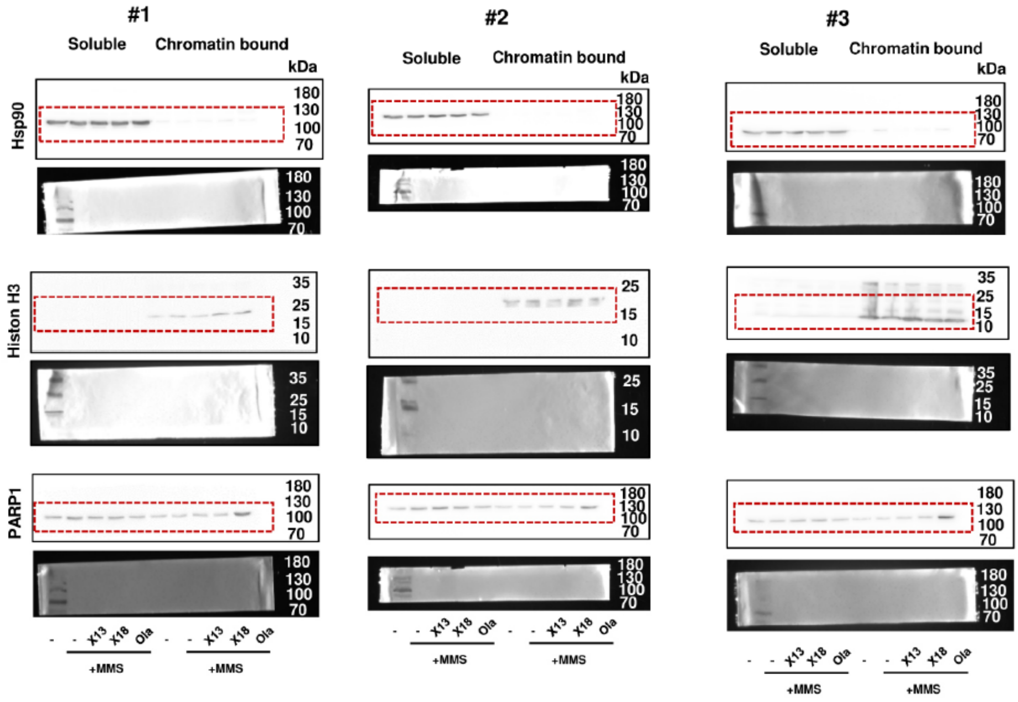

Figure S10. Uncropped Western blot images of Caco-2 cells shown in Figure 3B (#1) and two independent repetitions (#2 and #3).

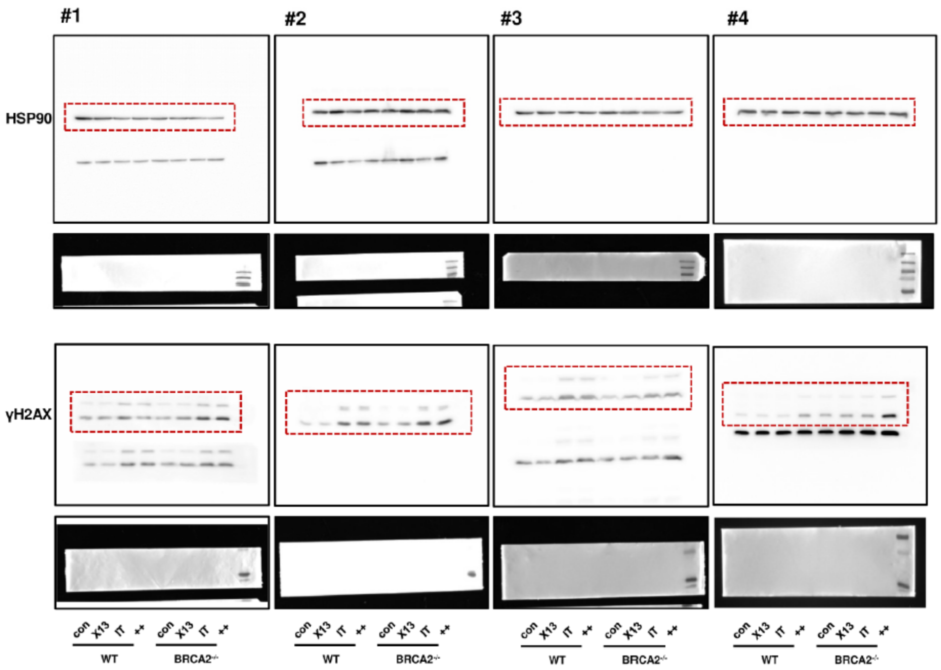

Figure S11. Uncropped Western blot images of HCT116 WT and BRCA2$^{-/-}$ cells shown in Figure 6C (#1) and three independent repetitions (#2–4).

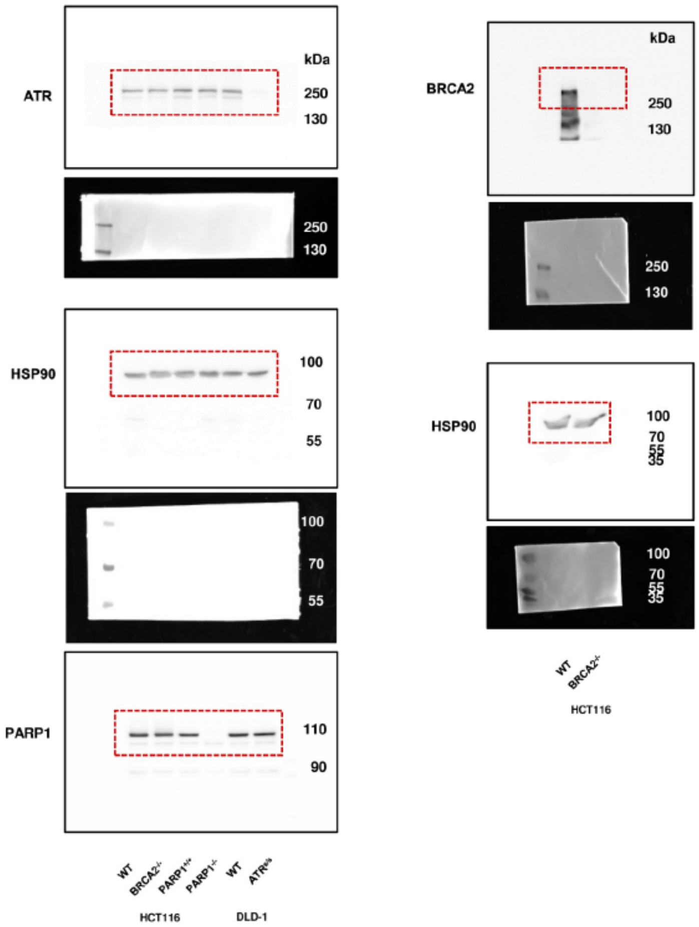

Figure S12. Uncropped Western blot images of HCT116 WT, HCT116 BRCA2$^{-/-}$, HCT116-PARP1$^{+/+}$, HCT116-PARP-1$^{-/-}$, DLD-1 WT, and DLD1 ATR$^{S/S}$ cells shown in Figure A5.

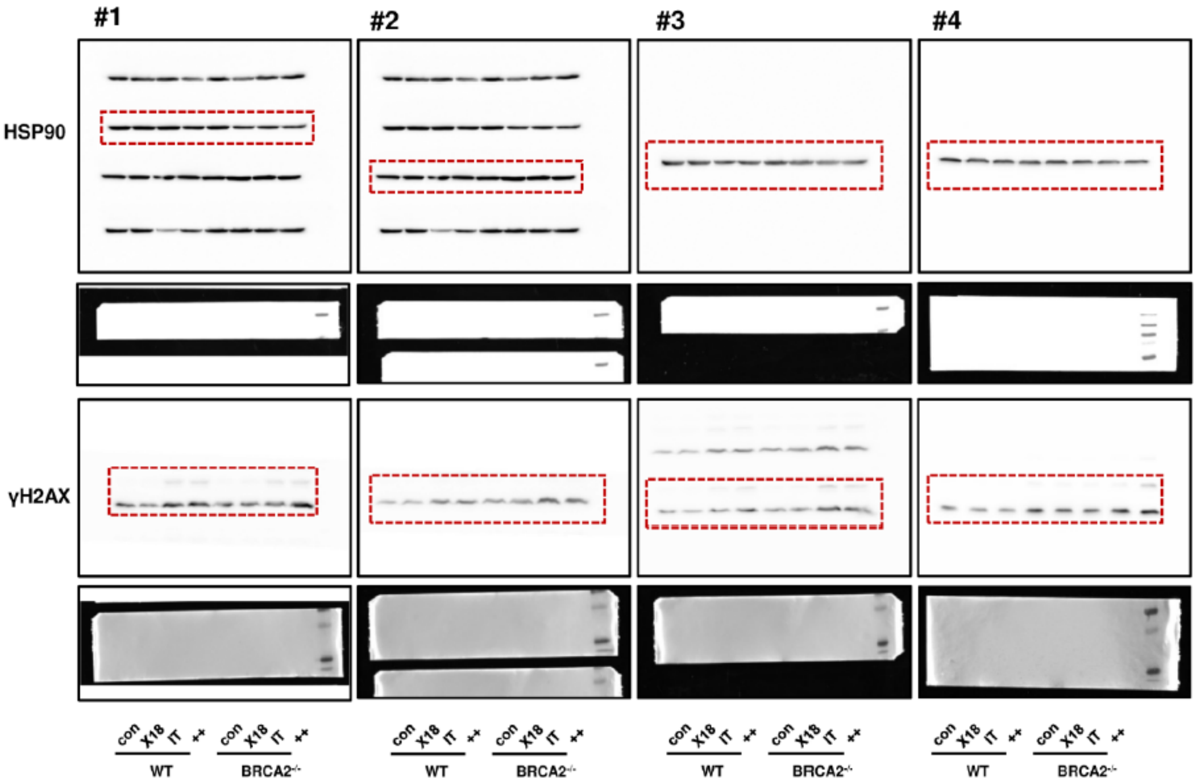

Figure S13. Uncropped Western blot images of HCT116 WT and BRCA2-/- cells shown in Figure A6C (#1) and three independent repetitions (#2–4).
